# Bimodal Antimicrobial Surfaces of Phytic Acid–Prussian Blue Nanoparticles–Cationic Polymer Networks

**DOI:** 10.1002/advs.202300354

**Published:** 2023-04-07

**Authors:** Xiaodong He, HuaJun Wu, Yan Wang, Yunjie Xiang, Kai Zhang, Xi Rao, En‐Tang Kang, Liqun Xu

**Affiliations:** ^1^ Chongqing Key Laboratory for Advanced Materials and Technologies of Clean Energies School of Materials and Energy Southwest University Chongqing 400715 P. R. China; ^2^ Department of Chemical and Biomolecular Engineering National University of Singapore Kent Ridge 117576 Singapore; ^3^ Key Laboratory of Laser Technology and Optoelectronic Functional Materials of Hainan Province College of Chemistry and Chemical Engineering Hainan Normal University Haikou 571158 P. R. China

**Keywords:** antibacterial, cationic polymers, photothermal, phytic acid, Prussian blue nanoparticles

## Abstract

Surface modification plays a pivotal role in tailoring the functionalities of a solid material. Introduction of antimicrobial function on material surfaces can provide additional protection against life‐threatening bacterial infections. Herein, a simple and universal surface modification method based on surface adhesion and electrostatic interaction of phytic acid (PA) is developed. PA is first functionalized with Prussian blue nanoparticles (PB NPs) via metal chelation and then conjugates with cationic polymers (CPs) through electrostatic interaction. With the aid of surface adherent PA and gravitation effect, the as‐formed PA–PB–CP network aggregates are deposited on the solid materials in a substrate‐independent manner. Synergistic bactericidal effects of “contact‐killing” induced by the CPs and localized photothermal effect caused by the PB NPs endow the substrates with strong antibacterial performance. Membrane integrity, enzymatic activity, and metabolism function of the bacteria are disturbed in contact with the PA–PB–CP coating under near‐infrared (NIR) irradiation. The PA–PB–CP modified biomedical implant surfaces exhibit good biocompatibility and synergistic antibacterial effect under NIR irradiation, and eliminate the adhered bacteria both in vitro and in vivo.

## Graphical abstract

The phytic acid‐functionalized Prussian blue nanoparticles (PA–PB NPs) and cationic polymers (CPs) form a large network of aggregates via electrostatic attraction. The PA–PB–CP coatings can deposit on various substrate surfaces by one‐step co‐deposition method, and the CPs and PB NPs show qualitative antibacterial performance to eliminate the adhered bacteria via “contact‐killing” and photothermal therapy.

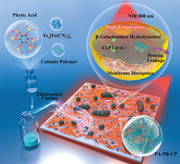

## 1 Introduction

Intrinsic surface properties in terms of wettability, biocompatibility, corrosion resistance, and antibacterial ability of a solid material are usually inadequate.^[^ ^1^ ^]^ Thus, surface modification of solid materials prior to application is essential to tailor the surface with desirable functionalities and properties.^[^ ^2^ ^]^ Various methods, including chemical vapor deposition (CVD),^[^ ^3^ ^]^ physical vapor deposition (PVD),^[^ ^4^ ^]^ thermal spray coating, electrodeposition coating, sol–gel coating, layer‐by‐layer assembly, and bio‐inspired coating,^[^ ^5^ ^]^ have been developed to tailor the surface morphology and chemical structure/composition of a solid material.^[^ ^6^ ^]^ However, many surface modification strategies have their advantages and disadvantages for particular applications. For example, CVD is suitable for industrial mass production, but it requires expensive equipment and could generate toxic gaseous byproducts. In general, surface modification strategies are chosen case‐by‐case, depending on the physical and chemical properties of the solid substrate, as well as the nature of deposited/tethered molecules. The ultimate goal of surface modification is to develop a simple and viable approach for introducing the desirable intriguing properties.

Surface modification strategies based on mussel‐inspired polydopamine (PDA)^[^ ^7^ ^]^ and tea stains‐inspired polyphenol^[^ ^8^ ^]^ have been widely explored. The PDA‐ and polyphenol‐based coatings can be deposited on a vast array of materials in a substrate‐independent manner. The reactive coatings could be further utilized to impart new functionalities to the solid materials via postmodification. The PDA‐based coatings gave dark‐brown coloration to the solid materials, while the deposition of tannic acid (TA) based polyphenol coatings required high salt conditions.^[^ ^9^ ^]^ It is thus of interest to explore alternative surface modification strategies to modulate the surface properties.

Phytic acid (PA, 1,2,3,4,5,6‐hexakis(dihydrogen phosphate)), a natural and innoxious plant constituent, is mainly found in wheat bran, wheat germ, rice bran, whole grains, legumes, oilseeds, and nuts.^[^ ^10^ ^]^ Owing to its phosphate‐rich characteristics, PA can bind strongly with many metal ions, such as Zn^2+^, Mg^2+^, Cu^2+^, and Fe^3+^, via metal chelation.^[^ ^11^ ^]^ Upon contact with the metal and metal oxide substrates, PA is readily adsorbed on their surfaces to form the phosphate‐based conversion coatings.^[^ ^12^ ^]^ PA coordinates with iron(III) ions to produce superhydrophilic coatings on a variety of substrates in a one‐step process.^[^ ^13^ ^]^ The PA‐grafted poly(vinyl alcohol) was deposited on the mild steel substrate to form a conversion layer of anticorrosive paint system.^[^ ^14^ ^]^ The PA‐functionalized SiO_2_ nanospheres were deposited on the metal meshes to fabricate robust superhydrophobic and superoleophilic surfaces.^[^ ^10c^ ^]^ As such, PA is a promising surface modifier for tailoring the surface properties.

Prussian blue (PB, Fe^III^_4_[Fe^II^(CN)_6_]_3_), one of the oldest synthetic coordination compounds, is biocompatible and nontoxic.^[^ ^15^ ^]^ PB has been approved by U.S. Food and Drug Administration (FDA) for treating internal radiation contamination.^[^ ^16^ ^]^ Due to charge transfer transition between Fe(II) and Fe(III), the PB‐based nanomaterials have strong optical absorbance in the near‐infrared (NIR) region and exhibit excellent photothermal performance on bacterial eradication and tumor ablation.^[^ ^17^ ^]^ PA was utilized to functionalize the UiO66–NH_2_ metal‐organic framework (MOF)^[^ ^18^ ^]^ and Ce–Zr–MOF.^[^ ^19^ ^]^ It would be interesting to modify PB with hydrophilic PA, leading to the formation of surface‐decorated PA–PB nanoparticles (NPs).

**Figure 1:**
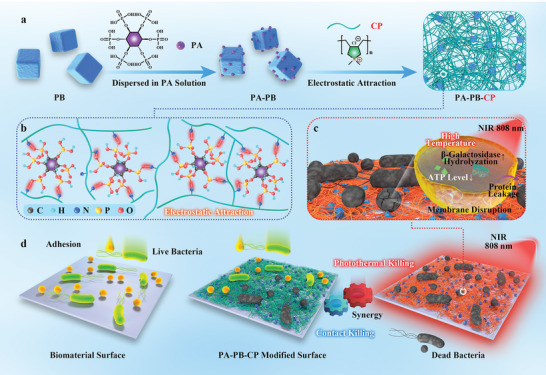
Design of PA–PB–CP network coating for antibacterial application. a) Formation of PA–PB NPs and PA–PB–CP network aggregates. b) The electrostatic attraction between PA and CP. c) Illustration of the plausible antibacterial mechanism of PA–PB–CP network coating. d) Schematic illustration of the “contact‐killing” and photothermal antibacterial ability of the PA–PB–CP network coating.

Polymers can promote rapid deposition of the desired molecules or NPs on solid materials through chain‐induced interactions and gravitational sedimentation.^[^ ^20^ ^]^ There are strong electrostatic interactions between PA and cationic polymers (CPs), such as chitosan,^[^ ^21^ ^]^ *ε*‐poly‐L‐lysine^[^ ^22^ ^]^ and polyethylenimine.^[^ ^23^ ^]^ PA on the PA–PB NPs could interact with CPs via electrostatic attraction, leading to the formation of PA–PB–CP network aggregates. Their gravitational deposition and concurrent surface adhesion of PA with the solid substrates could generate the PA–PB–CP coatings. In this work, PA‐induced rapid surface co‐deposition of PB NPs with four different types of CPs were studied in details. CPs could interact with the anionic bacterial cell membrane, resulting in cell wall permeabilization or pore formation, and leading ultimately to cell death.^[^ ^24^ ^]^ Since the CPs exhibit strong “contact‐killing” effects and the PB NPs possess excellent photothermal bactericidal ability, their synergistic antibacterial performance on the nonwoven fabric (NWF) for ventilation air cleaning was evaluated. The in vitro and in vivo antibacterial efficacy of the PA‐PB‐CP coatings on the biomedical implant surfaces were also assayed (**Figure** **1**).

## 2 Results and Discussion

**Figure 2:**
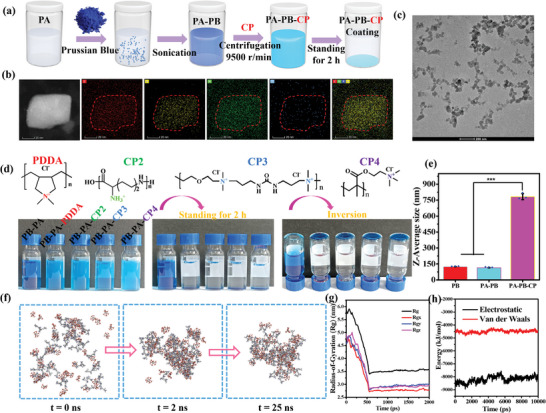
Ionic network coating formation and simulation. a) Schematic illustration of the formation of PA–PB NPs and their gravitational deposition in the presence of CP. b) EDS mapping and c) TEM image of the PA–PB NPs. d) The PA–PB NPs and PDDA (CP2, CP3, or CP4) solution mixtures. e) *Z*‐average sizes of the PB, PA–PB, and PA–PB–PDDA aggregate. f) Snapshots of the assembly of PA–PDDA at 0, 2, and 25 ns in the MD simulation. g) *R*_g_ (total and around axes) of PA–PDDA in the heating NPT conditions within 2 ns. h) Total electrostatic attraction and Van der Waals interaction energies of PA and PDDA. The error bars indicate the means ± SD (*n* ≥ 3); ^***^ *p* < 0.001.

PB NPs were dispersed in the PA solution, sonicated, and centrifuged to collect the supernatant (**Figure** **2**a). A stable aqueous dispersion of PA‐functionalized PB (PA–PB) NPs was obtained (Figure S1, Supporting Information). The energy dispersive X‐ray spectroscopy (EDS) elemental mappings of the PA–PB NPs are shown in Figure S2 (Supporting Information) and Figure 2b, while Figure 2c shows the transmission electron microscopy (TEM) image of the PA–PB NPs. The C (red), Fe (yellow), and N (green) species were homogeneously distributed throughout the NPs, while the P (blue) signal concentrated mainly in the outer layer. As shown in the scanning electron microscopy (SEM) images (Figure S3, Supporting Information), the surface morphology of PB NPs did not change appreciably before and after decorating with PA, and the average sizes of PA–PB NPs were measured to be 84.9 nm. Aqueous dispersions of both the PB and PA–PB NPs exhibit broad absorption peaks at about 685 nm with a strong adsorption tail extending into the NIR region (Figure S4, Supporting Information), indicating that they could serve as photothermal agents in the NIR region.

PA could associate with CPs via electrostatic attraction. Four different CPs, viz., poly(dimethyldiallylammonium chloride) (PDDA), *ε*‐poly‐L‐lysine (CP2), poly bis(2‐chloroethyl)ether‐*alt*‐1,3‐bis[3‐(dimethylamino)propyl]urea (CP3), and poly(2‐methacryloxyethyltrimethylammonium chloride) (CP4), were mixed with PA in the respective aqueous media. Their interactions and gravitational deposition were visualized, as the turbid mixtures became transparent gradually. In addition, soft and transparent coatings were formed on the bottom of glass vials (Figure S5, Supporting Information). Since PA and CPs could coalesce with each other, the assembly between PA–PB NPs and CPs was then studied. The *z*‐average sizes of PB and PA–PB NPs were about 120 nm. Upon the addition of PDDA, the *z*‐average size of PA–PB–PDDA aggregate increased to about 780 nm (Figure 2e). After the solution mixture was allowed to stand for 2 h, the PA–PB–PDDA suspension became clear, and a blue soft layer was formed on the bottom of inverted vial. Similar phenomenon was observed for the corresponding PA–PB–CP2, PA–PB–CP3, and PA–PB–CP4 solution mixtures (Figure 2d). However, CP alone cannot induce the gravitational deposition of PB NPs under the same condition (Figure S1, Supporting Information). These results confirm that interactions between PA and CPs allow the construction of PB‐embedded coatings. Furthermore, simulations of molecular dynamics (MD) were used to study the interactions of CP and PA in H_2_O.^[^ ^25^ ^]^ The MD simulation was performed under the NPT conditions, wherein the number of particles (*N*), pressure (*P*), and temperature (*T*) are all constant. The system was started with the initial instability at 273.15 K, and subjected to 1 ns of heating to reach 298.15 K. As shown in Figure 2f, the disordered PA and PDDA molecules tended to aggregate in aqueous solution. The radius of gyration (*R*_g_) gradually decreased, and reached the steady state at around 540 ps (Figure 2g). *R*_g_ is commonly used characterize the degree of molecular tightness. The decrease in *R*_g_ indicated PA and PDDA were tightly packed. The electrostatic attraction and Van der Waals interaction energies of PA and PDDA were tracked in the NPT process (Figure 2h). Similar MD simulations were also performed on CP2, CP3, and CP4. The results indicated that the electrostatic, Van der Waals, or hydrogen bonding interactions reinforced the PA–CP networks (Figures S6–S9, Supporting Information).

**Figure 3:**
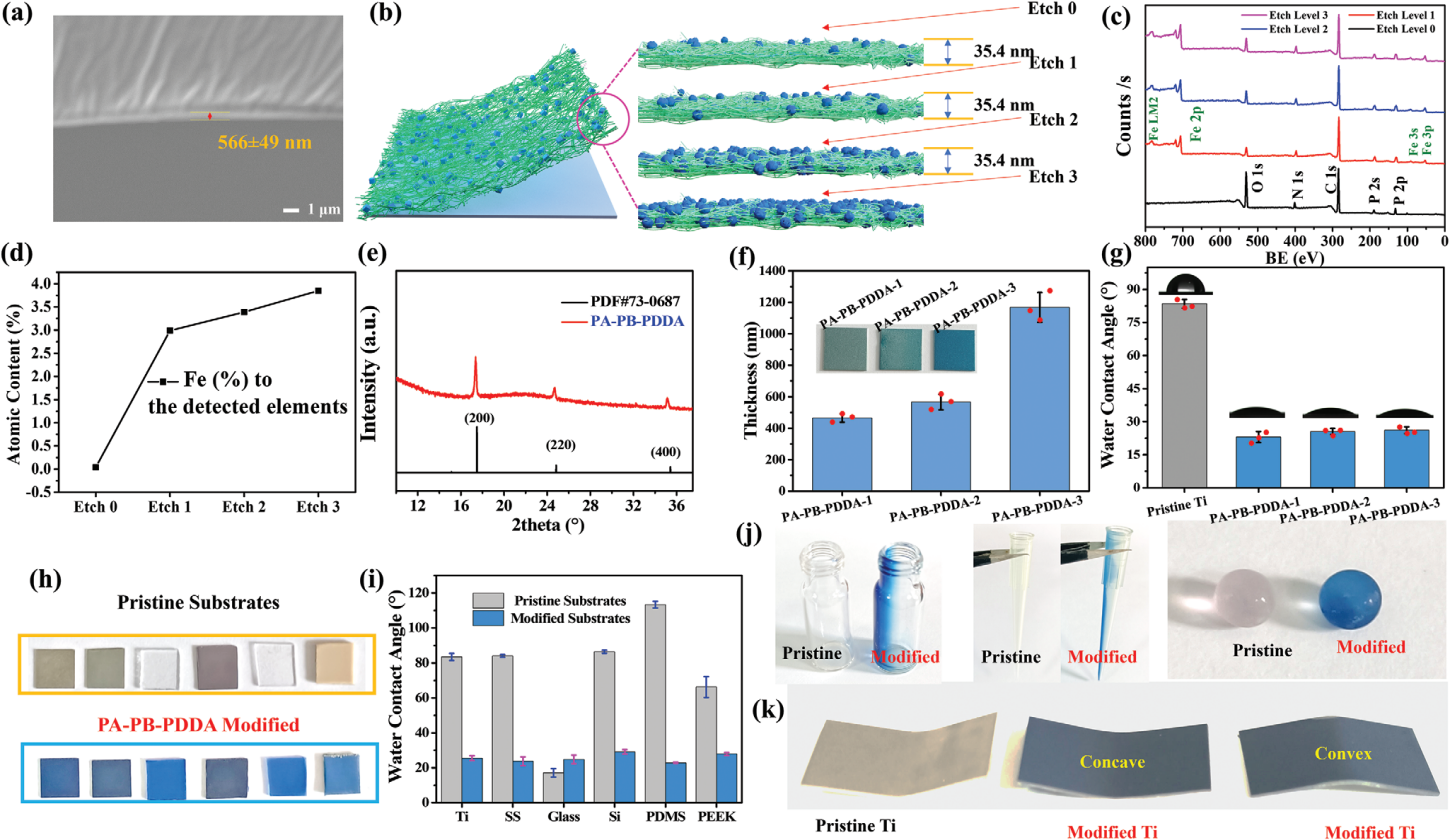
Surface deposition of the PA–PB–PDDA coatings. a) Cross‐sectional SEM image of the PA–PB–PDDA–coated PDMS substrate. b) Illustration of argon ion etching of the PA–PB–PDDA coating layer. c) XPS wide‐scan spectra of the PA–PB–PDDA coating after different etching cycles. d) XPS‐derived atomic contents (ratios) of Fe to C, N, O, P, and Fe at different etching levels. e) XRD spectrum of the PA–PB–PDDA coating. f) Thickness and g) water contact angles of PA–PB–PDDA coatings prepared by different PB contents. h) The photo images of Ti, SS, glass, Si, PDMS, and PEEK (from left to right) surfaces before and after deposition of the PA–PB–PDDA coating. i) The water contact angle values of the pristine and modified surfaces. The PA–PB–PDDA coated j) glass vial, pipette tip, silica gel, and k) curved Ti sheets.

Successful deposition of PA–PB–PDDA coatings was then verified by the X‐ray photoelectron spectroscopy (XPS) measurements (Figure S10, Supporting Information). The characteristic signals of the underlying substrates have either disappeared or attenuated, whereas the P 2p and N 1s signals, ascribed to the deposited PA and PDDA segments, were present in the XPS wide‐scan spectra of the modified substrates. The PB NPs were embedded in the PA and PDDA matrices, and their characteristic Fe signals were not readily discernible. Since the effective probing depth for an XPS measurement is less than 10 nm in an organic matrix,^[^ ^26^ ^]^ the PA–PB–PDDA coating was further analyzed by EDS which has a penetration depth of about 1 µm.^[^ ^27^ ^]^ The detection limit of EDS analysis is thus larger than the coating thickness of ≈566 nm, as measured by cross‐sectional view of SEM (**Figure** **3**a). As shown in the EDS mapping images (Figure S11, Supporting Information), the C, N, Fe, P, and O elemental signals are homogeneously distributed in the PA–PB–PDDA coating. Surface elemental molar ratio of the PA–PB–PDDA coating was analyzed using optical emission spectrometers with inductively coupled plasma (ICP–OES, Thermo Scientific iCAP PRO ICP–OES system). The surface P/Fe ratio was determined to be 7.67. Argon ion etching^[^ ^28^ ^]^ of the PA–PB–PDDA coating (0.59 nm s^−1^, 60 s, and 3 levels) was also performed to verify the presence of PB NPs (Figure 3c). The Fe 2p signal became visible in the coating after etching, and its atomic ratio increased with the increase in etching cycle (Figure 3d). The X‐ray diffraction (XRD) spectrum of the PA–PB–PDDA coating (Figure 3e) showed the peaks indexed at 2*θ* values of 17.4° (200), 24.7° (220), and 35.1° (400),^[^ ^29^ ^]^ consistent with the structure of PB NPs. The thickness of PA–PB–PDDA coating can be modulated by increasing the PB contents in the PA–PB–PDDA1, PA–PB–PDDA2, and PA–PB–PDDA3 coatings (Figure 3f). However, there is negligible change in the surface hydrophilicity of these substrates of varied PB contents, as shown in the water contact angle measurement (Figure 3g). The versatility of this coating strategy on various substrates, such as titanium (Ti), stainless steel (SS), glass, silicone (Si), polydimethylsiloxane (PDMS), and poly(ether ether ketone) (PEEK), and different shapes (glass vial, pipette tip, silica gel, and curved Ti sheet) were then investigated. As shown in Figure 3h, the color of substrate surfaces has changed in all cases after immersion in the solution mixture of PA–PB NPs and PDDA. The modified surfaces were hydrophilic due to the introduction of hydrophilic PA and PDDA components. The PA–PB–PDDA network aggregates can produce substrate‐independent coatings on various materials surfaces. The water contact angles of the substrates (except for the hydrophilic glass) decreased to 20–30° after deposition of the PA–PB–PDDA coating (Figure 3i). The PA–PB–PDDA coating can also be deposited on nonplanar surfaces, including the internal surfaces of glass vial and pipette tip, the outer surface of silica gel, as well as the curved Ti sheet (Figure 3j,k). Thus, the PA–PB–PDDA network aggregates can produce substrate‐independent coatings on various materials surfaces. The stability of the PA–PB–PDDA network coatings was assayed by vibration/agitation, sonication, heating, and immersion treatments. The physical and chemical treatments didn't change the appearance and integrity of the coatings (Figure S12, Supporting Information). The PA–PB–PDDA network coatings were soft, and their hardness on the Ti, glass, and PDMS substrates was between 0.1 and 0.5 GPa, as measured by a KLA G200 nanoindenter (Figure S13, Supporting Information).

**Figure 4:**
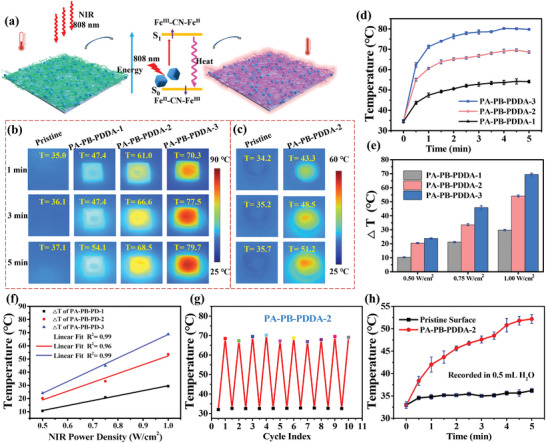
Photothermal effect of the PA–PB–PDDA coatings. a) Illustration of the energy conversion of the PA–PB–PDDA coating. The infrared images of b) PA–PB–PDDA coatings and c) PA–PB–PDDA‐2 coating in water under NIR (808 nm, 0.75 W cm^−2^) irradiation for different time. d) The time‐dependent temperature changes of the PDMS–PA–PB–PDDA surfaces under NIR (808 nm, 0.75 W cm^−2^) irradiation. e) Δ*T* of the PDMS–PA–PB–PDDA surfaces under NIR irradiation of different power intensities. f) Linear fitting of Δ*T* and NIR power density. g) The cyclic heating–cooling performance of the PA–PB–PDDA‐2 coating. h) The time‐dependent temperature changes of the pristine PDMS and PDMS–PA–PB–PDDA‐2 surfaces in 0.5 mL water under NIR (808 nm, 0.75 W cm^−2^) irradiation. The error bars indicate the means ± SD (*n* ≥ 3).

PB can generate heat under NIR irradiation for bacterial eradication and tumor ablation.^[^ ^30^ ^]^ The electronic transition between {Fe^III^[(t_2g_)^3^(e_g_)^2^]Fe^II^[(t_2g_)^6^]} and {Fe^II^[(t_2g_)^4^(e_g_)^2^]Fe^III^[(t_2g_)^5^]} results in NIR absorbance and photothermal effect in PB.^[^ ^31^ ^]^ After deposition of PA–PB–PDDA on the PDMS substrates, the photothermal effect of the modified substrates was evaluated (**Figure** **4**a). The pristine and PA–PB–PDDA1‐, PA–PB–PDDA2‐, and PA–PB–PDDA3‐coated PDMS surfaces were irradiated by NIR (808 nm, 0.75 W cm^−2^) to assay their photothermal effect using a Fluke TiS55 infrared thermal camera (Figure 4b,c). The temperatures of the pristine and modified PDMS surfaces increased with the increase in the irradiation time as well as the PB contents, and reached their respective plateaus after about 4 min (Figure 4d). The temperature of the PA–PB–PDDA1, PA–PB–PDDA2, and PA–PB–PDDA3 surface could reach to 54.1 °C (Δ*T*1 = 20.5 °C), 68.5 °C (Δ*T*2 = 33.6 °C), and 79.7 °C (Δ*T*3 = 54.1 °C), respectively, in 5 min at 0.75 W cm^−2^. The modified PDMS surfaces were also irradiated with NIR of different power densities (0.5–1.0 W cm^−2^). Δ*T* of the modified PDMS surfaces increased with the increase in NIR power density (Figure 4e), and they exhibited a linear relationship (Figure 4f). The photothermal stability of the PA–PB–PDDA2 coating was assayed by the repeated heating and cooling cycles (Figure S15, Supporting Information, and Figure 4g). The temperature‐induced attenuation was negligible after 10 cycles, indicating good photothermal cyclability and stability of the PA–PB–PDDA2 surface. Upon immersing the PA–PB–PDDA2 substrate in 0.5 mL water (Figure 4c), the solution temperature could reach to 51.2 °C after 5 min irradiation (Figure 4h).

Airborne particulates have adverse effects on human health, while filtration techniques are widely applied to mitigate their health and environmental effects.^[^ ^32^ ^]^ However, some microorganisms can grow on the filtration membranes under favorable temperature and humidity.^[^ ^33^ ^]^ Thus, the combination of photothermal antibacterial coating with NWF for air purification was explored (Figure S13a, Supporting Information). The NWF was soaked in the PA–PB–PDDA suspension for 2 h, followed by rinsing and drying. After surface functionalization, the modified NWF shows a light blue color (Figure S16b, Supporting Information), with the N, P, and Fe elements homogeneously distributed on the fiber surfaces (Figure S17, Supporting Information). The antibacterial performance of the PA–PB–PDDA‐coated NWF was assayed in an ingenious air purification system (Figure S16c, Supporting Information). The bacterial suspension was sprayed on the pristine and modified NWF. Compared to the pristine NWFs, the number of live bacteria on the modified NWF decreased significantly. This phenomenon can be attributed to the “contact‐killing” ability of the cationic PDDA segments on the modified NWF. Under NIR irradiation (808 nm, 0.75 W cm^−2^, 10 min), the pristine NWF exhibited negligible bactericidal effect against *Staphylococcus aureus* (*S. aureus*), while the modified NWF could kill all the adhered bacteria via the hyperthermal effect (Figure S16d, Supporting Information).^[^ ^34^ ^]^ After immersion in the bacterial suspension, the modified NWF killed most of the adhered bacteria, and eradicated the remaining bacteria (*S. aureus* and *Escherichia coli* (*E. coli*)) by photothermal effect (Figures S16e,f, and S18, Supporting Information). Furthermore, the PA–PB–PDDA‐coated NWF was tested for its cyclical bactericidal capability. After four cycles, the PA–PB–PDDA coating could still eliminate the adhered bacteria completely under NIR irradiation (Figures S16g and S19, Supporting Information).

**Figure 5:**
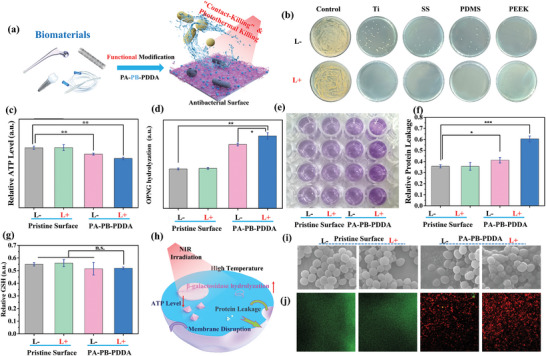
Antibacterial performance of PA–PB–PDDA coated biomaterial surfaces. a) Schematic illustration of the construction of “contact‐killing” and aPTT coating on biomaterial surfaces. b) The TSB agar plates inoculated with the detached *S. aureus* from the modified substrate surfaces. c) ATP activity, d) ONPG hydrolysis, e,f) BCA leakage and g) relative GSH level of *S. aureus* after incubation with the pristine and modified substrate surfaces in the presence/absence of NIR irradiation. h) Illustration of the plausible antibacterial mechanism. i) FESEM images of *S. aureus* adhered on the pristine and PA–PB–PDDA‐modified SS surfaces with (L+) or without (L−) NIR treatment (scale bar = 1 µm). j) CLSM images of MRSA biofilm on the pristine and PA–PB–PDDA‐modified PDMS surfaces (319.45 × 319.45 µm). The error bars indicate the means ± SD (*n* ≥ 3); ^**^ *p* < 0.01, ^***^ *p* < 0.001, and n.s. indicates no significance.

The insertion of biomedical prostheses and devices, such as orthopedic implants, dental implants, intravascular stents and catheters, has brought in the issue of implant‐associated infections. The Ti, SS, PDMS, and PEEK substrates are vital components of biomedical devices, but their surfaces are susceptible to bacterial colonization.^[^ ^35^ ^]^ The PA–PB–PDDA coatings were applied on their surfaces for “contact‐killing” and antibacterial photothermal therapy (aPTT) of the adhered bacteria (**Figure** **5**a). In the antibacterial adhesion assay, only a small number of viable *E. coli* and *S. aureus* adhered to the PA–PB–PDDA‐coated substrates. Under NIR illumination, all the bacteria on the modified surfaces were killed (Figure 5b and Figure S20, Supporting Information).

To elucidate the antibacterial mechanism of the PA–PB–PDDA coating, the adenosine triphosphate (ATP) activity, protein leakage, and *o*‐nitrophenyl‐*β*‐D‐galactoside (ONPG) hydrolysis activity of *S. aureu*s were evaluated.^[^ ^36^ ^]^ In comparison to the L− group of pristine substrates, approximately 10.6% and 17.8% decreases in ATP activity were observed on the PA–PB–PDDA L− and L+ groups, respectively (Figure 5c). In the ONPG hydrolysis assay, the produced *o*‐nitrophenol in the PA–PB–PDDA L− and L+ groups is significantly higher than that in the pristine substrate group (Figure 5d and Figure S21, Supporting Information). More ONPG could penetrate the more severely damaged bacterial membranes, and be hydrolyzed by *β*‐galactosidase. The protein leakage in the bacterial cells was assayed by bicinchoninic acid (BCA) protein assay kit (Figure 5e,f). Compared to the pristine substrate group, a larger amount of cytoplasmic proteins released from the PA–PB–PDDA L− group was observed. The difference became even more obvious after exposure to NIR irradiation. Glutathione (GSH) plays an important role in the bacterial antioxidant defense system.^[^ ^37^ ^]^ Ellman's assay was introduced to determine the possible oxidation of GSH by the photothermal effect of PA–PB–PDDA coating. The intracellular GSH level did not decrease significantly upon incubation with both PA–PB–PDDA L− and L+ groups (Figure 5g). These results indicated that the “contact‐killing” and aPTT effect of PA–PB–PDDA coating may have a strong effect on bacterial metabolic activity and membrane permeability (Figure 5h). The morphology of *S. aureus* on the pristine and modified SS surfaces was observed by field‐emission SEM (FESEM). The spherical structure of *S. aureus* on the pristine SS surface remained intact, and negligible change was observed after NIR exposure. However, the bacteria in contact with the PA–PB–PDDA coating had a deformed structure. Furthermore, most of the bacterial cells became wrinkled upon NIR irradiation (Figure 5i and Figure S22, Supporting Information). Thus, the “contact‐killing” and hyperthermal effect generated by the PA–PB–PDDA coating could have destructed the bacterial membranes, leading to bacterial death.^[^ ^38^ ^]^ These results are consistent with the membrane permeability assay measured by ONPG hydrolysis and BCA protein leakage. The eradication of methicillin‐resistant *S. aureus* (MRSA) biofilm on the pristine and modified SS surfaces was observed by confocal laser scanning microscope (CLSM, Figure 5j). The PA–PB–PDDA coating could effectively eradicate the MRSA biofilm, as strong red fluorescence can be observed on its surface in the presence and absence of NIR irradiation.

**Figure 6:**
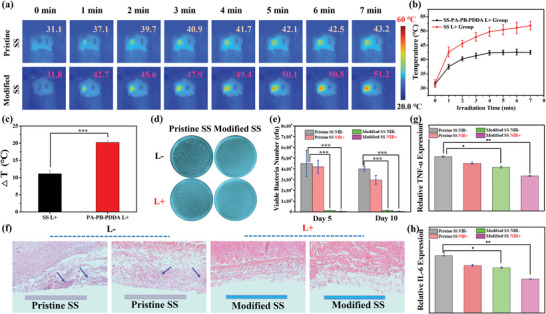
The subcutaneous infection model. a) The infrared thermal images of pristine SS and PA–PB–PDDA‐coated SS implants beneath the dorsal skin under NIR irradiation (808 nm, 1.5 W cm^−2^). b) The time‐dependent temperature changes of the pristine and modified SS‐embedded skin areas. c) Δ*T* of the pristine SS and SS‐PA–PB–PDDA surfaces. d) The TSB agar plates inoculated with the bacteria suspension detached from the pristine and modified SS substrates after implantation for 10 days at the same serial dilution. e) The number of viable bacteria on the pristine and modified SS implants in the presence (NIR+) and absence (NIR−) of irradiation. g) The H&E staining images of the skin tissues in contact with the pristine and modified SS implants. Relative expression of g) TNF‐*α* and h) IL‐6 of the infected tissues after 5 days according to a fluorescence‐based quantitative PCR assay. The error bars indicate the means ± SD (*n* ≥ 3); ^*^ *p* < 0.05, ^**^ *p* < 0.01, ^***^ *p* < 0.001, and n.s. indicates no significance.

Due to good penetration depth of the 808 nm NIR source,^[^ ^39^ ^]^ the aPTT performance of the PA–PB–PDDA coating was further evaluated in vivo in a subcutaneous infection model.^[^ ^40^ ^]^ The SD rats were implanted with the pristine SS and PA–PB–PDDA‐coated SS substrates beneath the dorsal skin, infected with *S. aureus*, and treated with and without NIR (Figure S23, Supporting Information). Under NIR irradiation (808 nm, 1.5 W cm^−2^), the temperatures of the skin areas embedded with the SS implants were recorded by an infrared thermal camera (**Figure** **6**a). As shown in Figure 6b, the temperature of the skin in contact with the modified SS implant increased with the increase in irradiation time, and reached to 51.2 °C after irradiation for 7 min. The temperature is effective for photothermal killing of pathogenic bacteria.^[^ ^41^ ^]^ In addition, there was a significant difference between the implanted pristine and modified SS surfaces (Figure 6c). After normal breeding for 5 and 10 days, the implants were retrieved, and the live bacteria on the surfaces were counted by the spread plate method (Figure 6d). The number of adhered bacteria on the modified SS substrate decreased significantly, in comparison to that on the pristine SS substrate. The number of bacteria on the SS‐PA–PB–PDDA surfaces was reduced further with the photothermal treatment (Figure 6e). The synergistic effect of “contact‐killing” and photothermal therapy endows the PA–PB–PDDA coating with good antibacterial efficacy in vivo. After implantation, the inflammatory responses of the skin tissues were investigated by hematoxylin‐eosin (H&E) staining assay (Figure 6f). Numerous neutrophils (indicated by blue arrows) infiltrated the skin tissues in contact with the infected pristine SS implants in the absence and presence of NIR irradiation. In the modified SS groups with and without NIR exposure, less inflammatory cells were observed, indicating the effective anti‐infection and immune‐evasion characteristics of the PA–PB–PDDA coating. Expression of inflammation factors in the infected issues, including TNF‐*α* and IL‐6, was tested after infection for 5 d.^[^ ^42^ ^]^ The PA–PB–PDDA coatings, both in the NIR+ and NIR− groups, were associated with the low expression of proinflammatory cytokine of TNF‐*α* and IL‐6 (Figure 6g,h). The PA–PB–PDDA coatings exhibited good antibacterial performance and anti‐inflammatory activity in the treatment in vivo. In addition, there was no pathological changes in the heart, liver, spleen, lung, and kidney in each group after implantation of the pristine and modified SS substrates (Figure S24, Supporting Information).

The biocompatibility of PA–PB–PDDA coating was further evaluated by the cell adhesion, cytotoxicity, and hemolysis assays. As shown in Figure S25 (Supporting Information), the PA–PB–PDDA coating did not prevent the adhesion for MC3T3‐E1 cells. The cytotoxicity of PA–PB–PDDA coating toward MC3T3‐E1 cells was assayed by the CCK‐8 method. The samples were immersed in minimum essential medium (MEM) for 1, 2, 3, and 4 days, and the extracts were cultured with the MC3T3‐E1 cells for 24 h. In comparison to the complete growth medium, the medium extracts exhibited negligible cytotoxicity toward MC3T3‐E1 cells (Figure S26, Supporting Information). The IL‐6 ELISA Kit was used to assess the effects of PA–PB–PDDA composite on the production of proinflammatory cytokines (IL‐6) in RAW264.7 cells. There was no significant difference on the released amount of IL‐6 between the control and PA–PB–PDDA groups (Figure S27, Supporting Information). The hemocompatibility of PA–PB–PDDA coating was further evaluated by the hemolysis assay (Figure S28, Supporting Information). The pristine and modified Ti substrates were immersed in the suspension of red blood cells (RBCs) at 37 °C for 2 h. The hemolytic rates of the pristine and modified Ti substrates were low, indicating that the PA–PB–PDDA coating exhibited good hemocompatibility.

## 3 Conclusion

A novel surface modification method for the construction of “contact‐killing” and photothermal antibacterial coatings on various substrates was developed. In comparison to the previously reported photothermal antibacterial coatings,^[^ ^43^ ^]^ the current strategy didn't require tedious processing. The PA‐functionalized PB NPs and CPs formed a large network of aggregates via electrostatic attraction, Van der Waals, or hydrogen bonding interactions. Surface adherence of the PA molecule and gravitational deposition of the as‐generated aggregates resulted in the formation of universal and robust coatings. The coatings could be deposited onto various substrate of different geometry, and were resistant to moderate chemical and physical treatments. The PA–PB–PDDA coating on the NWF surfaces could eliminate the airborne bacterial contaminants under NIR irradiation in an ingenious air purification model. The PA–PB–PDDA modified biomedical implant surfaces, including Ti, SS, PDMS, and PEEK, could prevent bacterial adhesion, due to the strong “contact‐killing” and photothermal therapy abilities of the coatings. In addition, the PA–PB–PDDA coated SS substrates exhibited good anti‐infection and immune‐evasion performance in a subcutaneous *S. aureus* infection model. The PA–PB–PDDA coating also showed good biocompatibility in the cytotoxicity and hemolysis assays. The developed coating strategy could be utilized to construct effective “contact‐killing” and photothermal therapy surfaces on solid materials for anti‐infection biomedical applications.

## 4 Experimental Section

All the method details are provided in Supporting Information.

## Conflict of Interest

The authors declare no conflict of interest.

## Supporting information

Supporting Information

## Data Availability

The data that support the findings of this study are available from the corresponding author upon reasonable request.

## References

[1] a) K. Wieszczycka , K. Staszak , M. J. Woźniak‐Budych , J. Litowczenko , B. M. Maciejewska , S. Jurga , Coord. Chem. Rev. 2021, 436, 213846;

[2] M. Mozetič , Materials 2019, 12, 441. 30709009 10.3390/ma12030441 PMC6384733

[3] X. Wang , P. J. Krommenhoek , P. D. Bradford , B. Gong , J. B. Tracy , G. N. Parsons , T. J. Luo , Y. T. Zhu , ACS Appl. Mater. Interfaces 2011, 3, 4180. 21985010 10.1021/am201082m

[4] T. Shirman , D. Freeman , Y. D. Posner , I. Feldman , A. Facchetti , M. E. van der Boom , J. Am. Chem. Soc. 2008, 130, 8162. 18529052 10.1021/ja8029784

[5] a) A. B. Asha , Y. Chen , R. Narain , Chem. Soc. Rev. 2021, 50, 11668; 34477190 10.1039/d1cs00658d

[6] a) B. Fotovvati , N. Namdari , A. Dehghanghadikolaei , J. Manuf. Mater. Process. 2019, 3, 28;

[7] a) X. Du , L. Li , J. Li , C. Yang , N. Frenkel , A. Welle , S. Heissler , A. Nefedov , M. Grunze , P. A. Levkin , Adv. Mater. 2014, 26, 8029; 25381870 10.1002/adma.201403709

[8] a) L. Q. Xu , K.‐G. Neoh , E.‐T. Kang , Prog. Polym. Sci. 2018, 87, 165;

[9] a) T. S. Sileika , D. G. Barrett , R. Zhang , K. H. A. Lau , P. B. Messersmith , Angew. Chem., Int. Ed. 2013, 52, 10766; 10.1002/anie.201304922 PMC3933447 24027124

[10] a) E. Feizollahi , R. S. Mirmahdi , A. Zoghi , R. T. Zijlstra , M. S. Roopesh , T. Vasanthan , Food Res. Int. 2021, 143, 110284; 33992384 10.1016/j.foodres.2021.110284

[11] a) C. Zhou , Z. Chen , H. Yang , K. Hou , X. Zeng , Y. Zheng , J. Cheng , ACS Appl. Mater. Interfaces 2017, 9, 9184; 28222262 10.1021/acsami.7b00412

[12] a) X. Tang , X. Zhang , Y. Chen , W. Zhang , J. Qian , H. Soliman , A. Qu , Q. Liu , S. Pu , N. Huang , G. Wan , Mater. Sci. Eng., C 2020, 108, 110487; 10.1016/j.msec.2019.110487 31923968

[13] L. Li , G. Zhang , Z. Su , Angew. Chem., Int. Ed. 2016, 55, 9093. 10.1002/anie.201604671 27377349

[14] Y. Zhao , X.‐H. Chen , J.‐M. Hu , Corros. Sci. 2021, 186, 109464.

[15] Q. Zheng , X. Liu , Y. Zheng , K. W. K. Yeung , Z. Cui , Y. Liang , Z. Li , S. Zhu , X. Wang , S. Wu , Chem. Soc. Rev. 2021, 50, 5086. 33634817 10.1039/d1cs00056j

[16] X. Wang , L. Cheng , Coord. Chem. Rev. 2020, 419, 213393.

[17] a) Y. Ren , H. Liu , X. Liu , Y. Zheng , Z. Li , C. Li , K. W. K. Yeung , S. Zhu , Y. Liang , Z. Cui , S. Wu , Cell Rep. Phys. Sci. 2020, 1, 100245;

[18] J. Zhang , Z. Li , L. Zhang , Y. Yang , D.‐Y. Wang , ACS Sustainable Chem. Eng. 2020, 8, 994.

[19] S. Yan , B. Luo , J. He , F. Lan , Y. Wu , J. Mater. Chem. B 2021, 9, 1811. 33503098 10.1039/d0tb02517h

[20] a) Y. Du , W.‐Z. Qiu , Z. L. Wu , P.‐F. Ren , Q. Zheng , Z.‐K. Xu , Adv. Mater. Interfaces 2016, 3, 1600167;

[21] J.‐Y. Yang , Y. Xia , J. Zhao , L.‐F. Yi , Y.‐J. Song , H. Wu , S.‐Y. Guo , L.‐J. Zhao , J.‐R. Wu , Chin. J. Polym. Sci. 2020, 38, 1294.

[22] X. He , J. Zhang , L. Xie , G. Sathishkumar , C. Li , X. Rao , J. Zhao , K. Zhang , R. Wang , E.‐T. Kang , L. Xu , Chem. Eng. J. 2022, 440, 135917.

[23] a) J. Wang , H. Guan , Q. Han , S. Tan , Q. Liang , M. Ding , ACS Biomater. Sci. Eng. 2019, 5, 2740; 33405606 10.1021/acsbiomaterials.9b00074

[24] D. J. Phillips , J. Harrison , S.‐J. Richards , D. E. Mitchell , E. Tichauer , A. T. M. Hubbard , C. Guy , I. Hands‐Portman , E. Fullam , M. I. Gibson , Biomacromolecules 2017, 18, 1592. 28365981 10.1021/acs.biomac.7b00210 PMC5435458

[25] J. J. Zhou , M. Penna , Z. X. Lin , Y. Y. Han , R. P. M. Lafleur , Y. J. Qu , J. J. Richardson , I. Yarovsky , J. V. Jokerst , F. Caruso , Angew. Chem., Int. Ed. 2021, 60, 20225. 10.1002/anie.202106316 PMC8405577 34258845

[26] S. J. Kerber , T. L. Barr , G. P. Mann , W. A. Brantley , E. Papazoglou , J. C. Mitchell , J. Mater. Eng. Perform. 1998, 7, 334.

[27] B. D. Ratner , D. G. Castner , Surface Analysis ‐ The Principal Techniques, 2nd Edition (Eds: J. C. Vickerman , I. S. Gilmore ), Wiley, Weinheim 2009 Ch. 3, p. 47.

[28] a) C. Miot , E. Husson , C. Proust , R. Erre , J. P. Coutures , J. Eur. Ceram. Soc. 1998, 18, 339;

[29] P. C. Pandey , S. Shukla , R. J. Narayan , Nanomaterials 2021, 11, 1145. 33925050 10.3390/nano11051145 PMC8146005

[30] L. G. Ning , P. Liu , B. Wang , C. M. Li , E. T. Kang , Z. S. Lu , X. F. Hu , L. Q. Xu , J. Colloid Interface Sci. 2019, 549, 72. 31022525 10.1016/j.jcis.2019.04.050

[31] a) F. S. Hegner , J. R. Galan‐Mascaros , N. Lopez , Inorg. Chem. 2016, 55, 12851; 27989203 10.1021/acs.inorgchem.6b02200

[32] a) M. Lan , S. Zhao , W. Liu , C. S. Lee , W. Zhang , P. Wang , Adv. Healthcare Mater. 2019, 8, 1900132; 10.1002/adhm.201900132 31067008

[33] Q. Pan , S. Zhang , R. Li , Y. He , Y. Wang , J. Mater. Chem. B 2019, 7, 2948.

[34] Y. Wang , T. Wei , Y. Qu , Y. Zhou , Y. Zheng , C. Huang , Y. Zhang , Q. Yu , H. Chen , ACS Appl. Mater. Interfaces 2020, 12, 21283. 31709795 10.1021/acsami.9b17581

[35] a) W. Feng , N. Liu , L. Gao , Q. Zhou , L. Yu , X. Ye , J. Huo , X. Huang , P. Li , W. Huang , J. Mater. Sci. Technol. 2021, 69, 188;

[36] Y. A. Li , X. M. Liu , B. Li , Y. F. Zheng , Y. Han , D. F. Chen , K. W. K. Yeung , Z. D. Cui , Y. Q. Liang , Z. Y. Li , S. L. Zhu , X. B. Wang , S. L. Wu , ACS Nano 2020, 14, 8157. 32585104 10.1021/acsnano.0c01486

[37] W. Y. Yin , J. Yu , F. T. Lv , L. Yan , L. R. Zheng , Z. J. Gu , Y. L. Zhao , ACS Nano 2016, 10, 11000. 28024334 10.1021/acsnano.6b05810

[38] R. M. Zhang , C. Li , W. Liu , Y. P. Huang , B. Wang , K. M. Xiao , P. K. Wong , L. Q. Ye , Environ. Sci.: Nano 2021, 8, 1446.

[39] a) Y. C. He , Z. Li , C. Cong , F. Ye , J. Y. Yang , X. W. Zhang , Y. Yuan , Z. H. Ma , K. Q. Zhang , Y. Lin , L. Z. Zheng , X. J. Liang , D. W. Gao , ACS Nano 2021, 15, 10488; 34018736 10.1021/acsnano.1c03048

[40] Y. Q. Zhao , Y. J. Sun , Y. D. Zhang , X. K. Ding , N. N. Zhao , B. R. Yu , H. Zhao , S. Duan , F. J. Xu , ACS Nano 2020, 14, 2265. 32017535 10.1021/acsnano.9b09282

[41] G. C. Qing , X. X. Zhao , N. Q. Gong , J. Chen , X. L. Li , Y. L. Gan , Y. C. Wang , Z. Zhang , Y. X. Zhang , W. S. Guo , Y. Luo , X. J. Liang , Nat. Commun. 2019, 10, 4336. 31551496 10.1038/s41467-019-12313-3 PMC6760232

[42] a) J. J. Ye , L. F. Li , R. N. Hao , M. Gong , T. Wang , J. Song , Q. H. Meng , N. N. Zhao , F. J. Xu , Y. Lvov , L. Q. Zhang , J. J. Xue , Bioact. Mater. 2023, 21, 284; 36157247 10.1016/j.bioactmat.2022.08.026 PMC9478498

[43] Y. W. Ren , H. P. Liu , X. M. Liu , Y. F. Zheng , Z. Y. Li , C. Y. Li , K. W. K. Yeung , S. L. Zhu , Y. Q. Liang , Z. D. Cui , S. L. Wu , Cell Rep. Phys. Sci. 2020, 1, 100245.

