# Supplementary material for: Bimodal Antimicrobial Surfaces of Phytic Acid–Prussian Blue Nanoparticles–Cationic Polymer Networks
Source: Adv Sci (Weinh). 2023 Apr 7;10(16):2300354. doi: 10.1002/advs.202300354 (PMC10238204; doi:10.1002/advs.202300354)
Supplement: Supplementary file 1 — Supporting Information [file ADVS-10-2300354-s001.pdf]

ADVANCED SCIENCE
Open Access

Supporting Information

for *Adv. Sci.*, DOI 10.1002/advs.202300354

Bimodal Antimicrobial Surfaces of Phytic Acid–Prussian Blue Nanoparticles–Cationic Polymer Networks

Xiaodong He, HuaJun Wu, Yan Wang, Yunjie Xiang, Kai Zhang, Xi Rao, En-Tang Kang and Liqun Xu**

Supporting Information

Bimodal Antimicrobial Surfaces of Phytic Acid-Prussian Blue Nanoparticles-Cationic Polymer Network

Materials: poly(dimethyldiallylammonium chloride) (PDDA, M_w, 200,000-350,000, 20 wt%), was purchased from Aladdin Biochemical Technology Co., Ltd. (Shanghai, China). CP2: ε-poly-L-lysine (M_w, 3,600-4,300), produced by the filamentous bacterium *Streptomyces albulus*, was kindly provided by Xu Hong's group of NanJing Tech University. CP3: poly bis(2-chloroethyl)ether-alt-1,3-bis[3-(dimethylamino)propyl]urea and CP4: poly(2-methacryloxyethyltrimethylammonium chloride) were purchased from Macklin Biochemical Co., Ltd. (Shanghai, China). Phytic acid (PA) (70% in water), $Fe^{III}_4[Fe^{II}(CN)_6]_3$ (PB, Product No.: C21521) and all other reagents were purchased from J&K Scientific Ltd. (Beijing, China), Sigma-Aldrich Chemical Co. (Shanghai, China), Adamas Reagent Co., Ltd. (Shanghai, China) and Sangon Biotech Co., Ltd. (Shanghai, China). *E. coli* (Type: CMCC 44102) and *S. aureus* (Type: CMCC 26003) were purchased from the National Center for Medical Culture Collection. MRSA (Type: ATCC 33592) was obtained from the American Type Culture Collection.

Preparation of PA-PB solution: The PB powder was placed in agate mortar and manually ground for 10 min. The fine powders (19.2, 25.6 and 32 mg) were mixed with 32 mL of the PA aqueous solution (1 mg/mL). The mixtures were sonicated for 3 h, and centrifuged at 9500 rpm for 10 min. The supernatants were obtained as the PA-PB-1, PA-PB-2, PA-PB-3 solutions, respectively. The PB concentrations in the PA-PB-1, PA-PB-2 and PA-PB-3 samples were measured by ICP-OES to be 0.086, 0.11 and 0.15 mg/mL, respectively. Similar procedure was employed to prepare the

WILEY-VCH

PB solution, without the addition of PA.

Preparation and surface deposition of the PA-PB-CP suspension: The PA-PB-CP was obtained by mixing 5 mL PA-PB solution and 1 mL CP (PDDA, CP2, CP3 or CP4) aqueous solution (2 mg/mL). The PA-PB-PDDA-coated substrates (Ti, SS, Glass, Si, PDMS, PEEK and NWF) were obtained by immersing the substrates into the PA-PB-PDDA suspension for 2 h. The surface modified substrates were washed with deionized water thoroughly and dried at room temperature.

Characterization: The UV-visible absorption spectroscopy was performed in the wavelength range between 300–900 nm on a Shimadzu UV-2550 spectrophotometer. The hydrodynamic sizes of the PB, PA-PB and PA-PB-PDDA aggregates were measured on a Nano ZS90 Zetasizer (Malvern Instruments, UK). The XRD pattern was recorded on a Shimadzu XRD-7000 diffractometer in the 2θ range from 10 to 40°. Surface compositions of the substrates before and after functionalization were evaluated by XPS measurements on a Thermo Scientific ESCALAB 250Xi photoelectron spectrometer. TEM images were obtained from a FEI Talos F200S TEM equipped with SuperX-EDS. The morphologies of coating surfaces and bacteria were observed under FESEM (JSM-7800F, JEOL Ltd., Tokyo, Japan). The static water contact angles of the substrates were measured on a JC2000D goniometer.

Computational methods: The molecular structure of PA was optimized based on DFT by using the Gaussian 09 packages at B3LYP/6-31G(d,p) level. The optimized structure was subjected to the frequency calculation to confirm no negative frequency. First, four CP's with polymerization degrees of 5 were constructed. Then, periodic annealing of the CP was performed to obtain a more stable structure for further calculation. The PA and CP (CP1, CP2, CP3 or CP4) water boxes were constructed in this work. Molecular dynamics (MD) simulations were carried out for the four systems. After energy minimization with the steepest descent algorithm, MD

WILEY-VCH

simulations were performed under NPT conditions with a 2 fs time step. The system was started with the initial instability at 273.15 K, and subjected to 1 ns of heating to reach 298.15 K. In subsequent simulations, the temperature was kept at 298.15 K with the V-rescale thermostat and a 0.2 ps scaling constant.[1] The isotropic Berendsen barostat was introduced to keep the pressure at 1 bar during the simulation, and the time constant for pressure relaxation was set to 0.5 ps.[2] Electrostatic and Van der Waals interactions were treated by means of the particle-mesh Ewald (PME) method with a real-space cutoff of 1.0 nm.[3] Periodic boundary conditions were applied in all three dimensions. All MD simulations were performed in Gromacs 5.0.[4] The graphics of MD results were obtained using the VMD software (version 1.9.3).[5]

Photothermal antibacterial properties of PA-PB-PDDA-coated NWF: *E. coli* and *S. aureus* were cultured overnight and diluted with phosphate-buffered saline (PBS) to a concentration of about 1×10^7 colony forming units (CFU)/mL. One mL of bacterial suspension was co-cultured with the pristine and surface functionalized NWFs (10 mm × 10 mm) in a 24-well plate. After incubation at 37 ^{o}C for 2 h, the bacterial suspension was discharged. The pristine and modified NWFs were irradiated with a NIR source (808 nm, 0.75 W/cm^2) for 10 min. Then, the pristine and modified NWFs were washed with PBS thrice, and sonicated in 3 mL of PBS for 8 min. The number of adhered bacteria on the pristine and modified NWFs was counted by the spread plate method.

To evaluate the air purification performance of pristine and PA-PB-PDDA-coated NWFs, an ingenious filtration system was used. The pristine and modified NWFs were fixed on the ventilator scoop, and the airborne contaminants containing *S. aureus* were introduced by mist spraying for 10 times. After 30 min, the pristine and modified NWFs were irradiated by NIR (808 nm, 0.75 W/cm^2) for 10 min, washed with 5 mL PBS, and sonicated for 6 min to detach the adhered bacteria. The collected bacterial suspension was diluted and spread on the TSB-agar plates. The bacterial

colonies on the TSB-agar plates were counted after culturing at 37 ^{o}C overnight.

FESEM Observation: The pristine and PA-PB-PDDA-coated SS (stainless steel) substrates were cultured with 1 mL bacterial suspension (1×10^7 CFU/mL) in TSB medium for 24 h. The SS and SS-PA-PB-PDDA substrates were treated with NIR (L+) or kept in the dark (L-) for 10 min. The substrates were washed with PBS thrice. The adhered bacteria were fixed by glutaraldehyde (3 wt%), and dehydrated using the ethanol gradients (25%, 50%,75% and 100%, v/v). Each ethanol dehydration step was performed for 15 min. The bacterial morphologies on the SS and SS-PA-PB-PDDA surfaces were observed by FESEM.

ONPG hydrolysis, ATP activity and BCA leakage assays: In the ONPG hydrolysis assay, *S. aureus* was cultured with pristine Ti and Ti-PA-PB-PDDA substrates in a 24-well plate for 5 h (*S. aureus*, 10^7 cfu/mL). The NIR+ group was irradiated by NIR (808 nm, 0.75W/cm^2) for 10 min. Five hundred μL of ONPG agent (ONPG assay Kit) was added into each well, and the plate was incubated at 37 ^{o}C for another 12 h. The OD value of ONPG agent was measured at 420 nm.

In the ATP activity assay, similar culture procedures were applied. After that, the substrates were sonicated in PBS for 7 min. The retrieved bacteria suspension and the bacterial suspension in well were combined and centrifuged, and the collected bacterial cake was sonicated in lysis buffer by using the 3 s-on/5 s-off setting of a cell disrupter. The sonicated bacteria suspension was transferred to a 96-well plate for ATP Assay Kit (S0026, Beyotime) measurement on a microplate reader.

In the protein leakage assay, after similar culture procedures, the working solution (Micro BCA Protein Assay Kit, Thermo Scientific) was added into each well for OD measurement.

In vivo antibacterial performance of PA-PB-PDDA coating in a subcutaneous implantation model: The rat subcutaneous infection model was performed to evaluate

the photothermal therapy performance of the PA-PB-PDDA-coated SS substrates. All the animal assays were carried out in accordance with the animal experiment guidelines ratified by the University Committee on Use and Care of Animals (UCUCA) at Southwest University (Approval No.: IACUC-20211115–02). All the experiments were expressed as mean values ± standard and Student's t-test were used for analyzing the significance. The SD rats (200-220g) were divided into 4 groups to receive the pristine SS and SS-PA-PB-PDDA substrates with and without the NIR irradiation. For the implant operation, the rats were anesthetized, and the dorsal section of the rat was shaved and sterilized. The skin was then incised, followed by the implantation of the pristine SS and SS-PA-PB-PDDA substrates (10 mm × 10 mm) beneath the skin. The implants were inoculated with 15 μL *S. aureus* suspension at a concentration of 1×10^7 CFU/mL, and the skin were stitched. After 5 and 10 days, the rats were sacrificed and the implants were retrieved. The spread plate method was used to determine the adhered bacteria on the implants. Histological observations of the heart, liver, spleen, lung, kidney and skin tissue of the healthy and implanted rats were evaluated by the H&E staining.

PCR test: The SD rats were euthanized, and the skin tissues in contact with the implants were collected and stored in the RNAsolid (Wuhan Servicebio, Cat. No.: G3019) RNA stabilization solution. One mL of RNA extract was added to a homogenate tube, and the tube was put on ice for pre-cooling. One hundred mg of tissue was added into the homogenate tube, and smashed by grinding machine fully until no visible tissue block. The solution mixtures were centrifuged at 12,000 rpm for 10 min to obtain the supernatant. Two hundred and fifty μL of trichloromethane was added into the centrifuge tube, and the tube was inverted for 15 s, vortexed and stand for 3 min. The solution mixtures were centrifuged at 12,000 rpm and 4 ℃ for 10 min . Four hundred μL of supernatant was transferred to a new centrifuge tube and 320 μL (0.8 times of the supernatant) of isopropyl alcohol was added. The tube was vortexed and stored at -20 ℃ for 15 min. The mixture was centrifuged at 12,000 rpm and 4 ℃

WILEY-VCH

for 10 min. The liquid was discarded, and 1.5 mL of 75% ethanol was added to wash the precipitate. The solution mixture was centrifuged at 12000 rpm and 4 °C for 5 min, and the supernatant was discarded. The centrifuge tube was placed in a biosafety cabinet, and air-dried for 3 min. The collected RNA was dissolved by adding 15 μL of Nuclease-free water, and incubated at 55 °C for 5 min. Concentration of RNA was determined on a NanoDrop 2000 spectrophotometer and adjusted to 100-500 ng/μL, if applicable. RNA quality of all samples was measured using a fluorescence-based quantitative PCR with reverse transcription (CFX, Bio-rad).

Cell adhesion and cytotoxicity of the PA-PB-PDDA coating: The MC3T3-E1 cells (5000 cells/well) were cultured in the pristine and PA-PB-PDDA-deposited wells of 24-well plates for 24 h. The cells were stained by Cell Plasma Membrane Staining Kit with DiO (Green Fluorescence, 3,3'-dioctadecyloxacarbocyanine perchlorate). The cytotoxicity of Ti-PA-PB-PD substrates was tested against MC3T3-E1 cells using the Cell Counting Kit-8 (CCK-8) assay. The sample was placed into the well of a 24-well plate, and 1 mL of Minimum Essential Medium (MEM) was added into each well. After immersion for 1, 2, 3 and 4 days, the extracted media were cultured with cells for 24 h. Ten μL of CCK-8 solution was added into each well, and the plate was cultured for another 2 h. The OD's at 450 nm of three parallel samples were measured and the average value was calculated.

Statistical analysis: Statistical data are presented as mean values ± SD. Statistical analysis was performed using one sample t-test by Origin Pro 9.1 software. All statistical data were collected from experiments with three replicates ($n = 3$). The alpha value is 0.05, the P value *$p < 0.05$, **$p < 0.01$, ***$p < 0.001$, and n.s. indicates no significance.

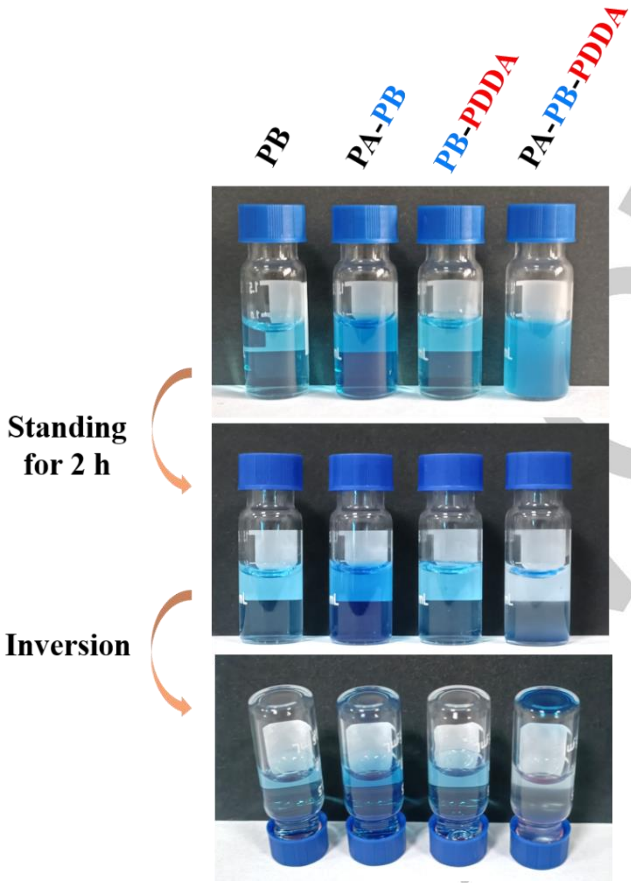

Figure S1. The image of PB, PA-PB, PB-PDDA and PA-PB-PDDA suspensions after standing for 2 h and inversion of the vials.

WILEY-VCH

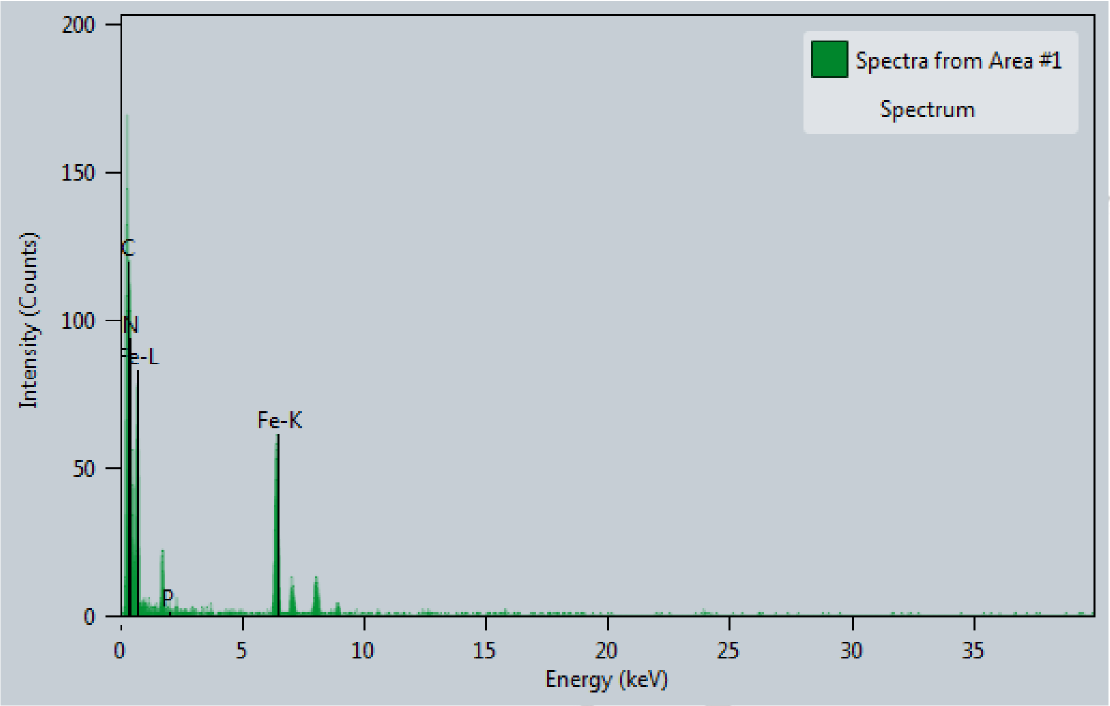

Figure S2. The EDS spectrum of the PA-PB NPs in Figure 2b.

WILEY

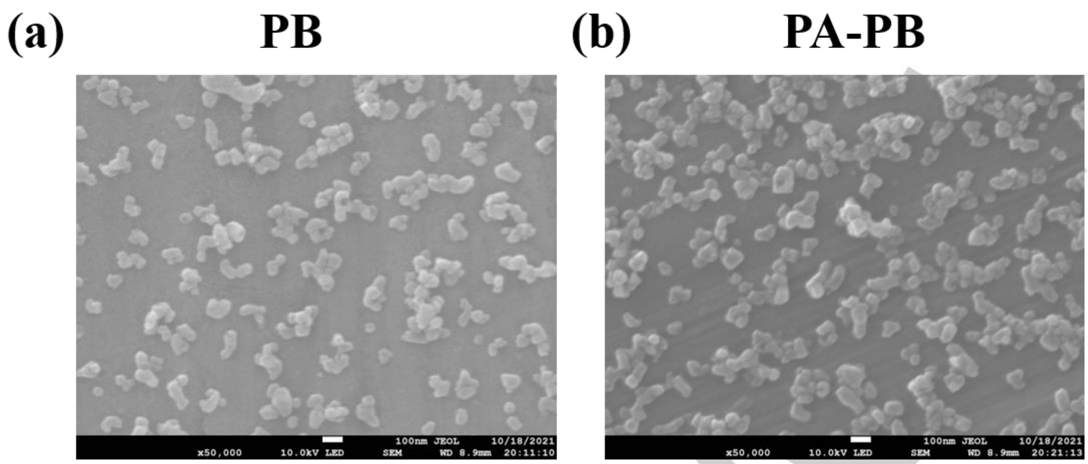

Figure S3. SEM images of PB and PA-PB NPs.

WILEY-VCH

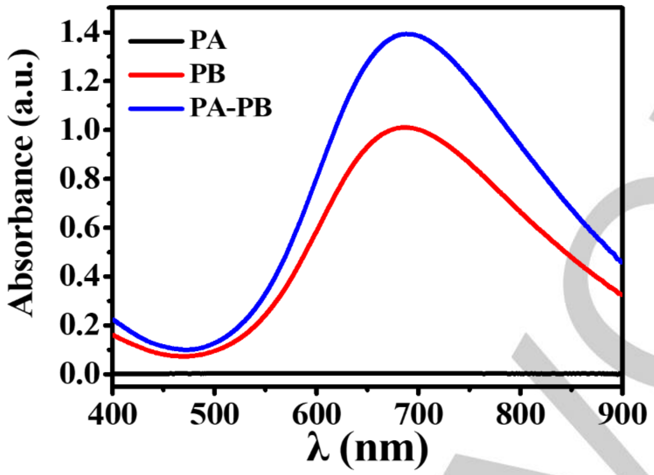

Figure S4. UV-visible absorption spectra of the PA, PB and PA-PB NPs.

WILEY-VCH

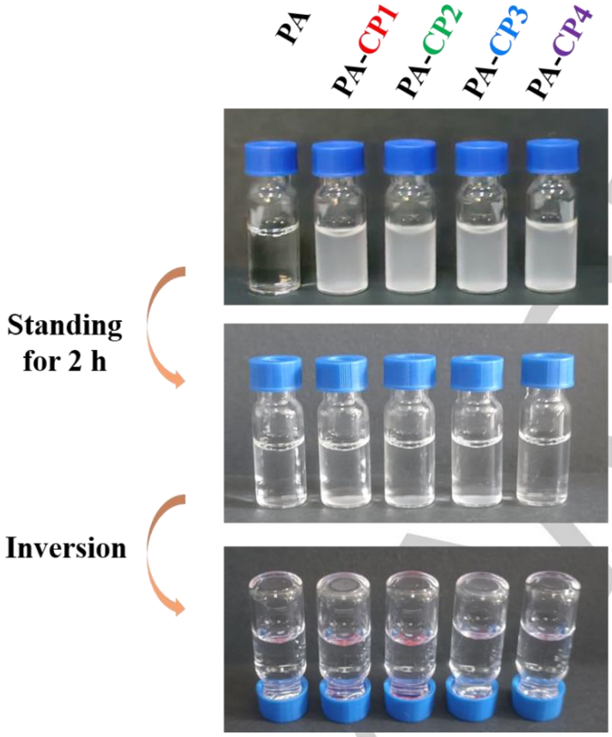

Figure S5. The solution mixtures of PA and PDDA (CP2, CP3 and CP4) after standing for 2 h and being inverted. The turbid solution mixtures became clear after standing, and transparent layers were formed on the bottom of inverted vials.

WILEY-VCH

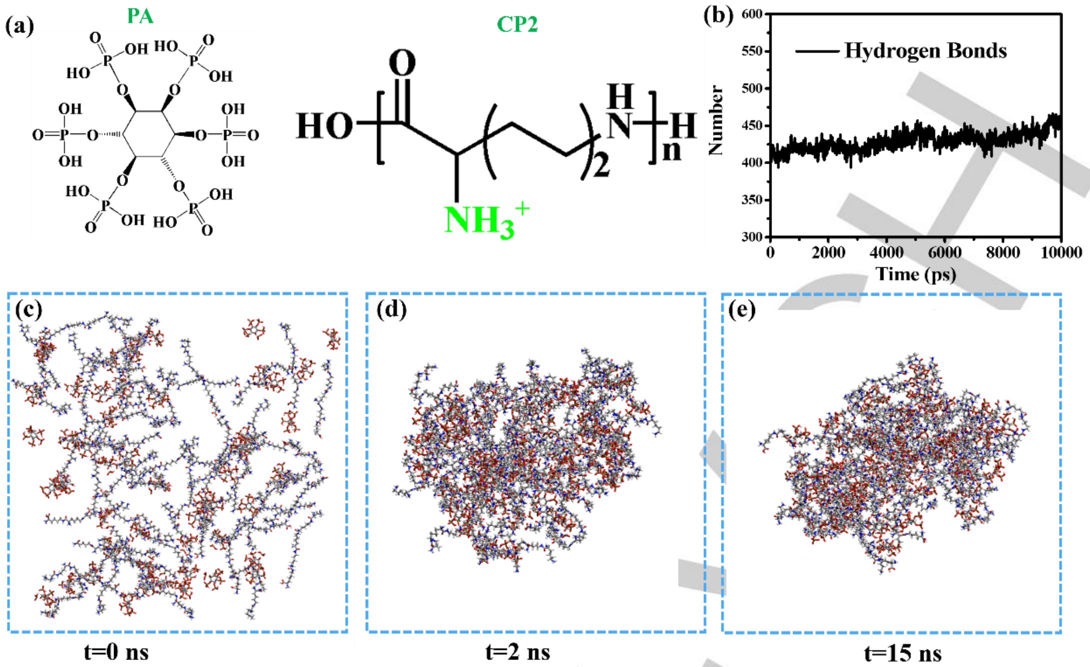

Figure S6. (a) Chemical structures of PA and CP2. (b) Number of hydrogen bonds of PA and CP2. Snapshots of the assembly of PA-CP2 at (c) 0, (d) 2 and (e) 15 ns in the MD simulation.

WILEY-VCH

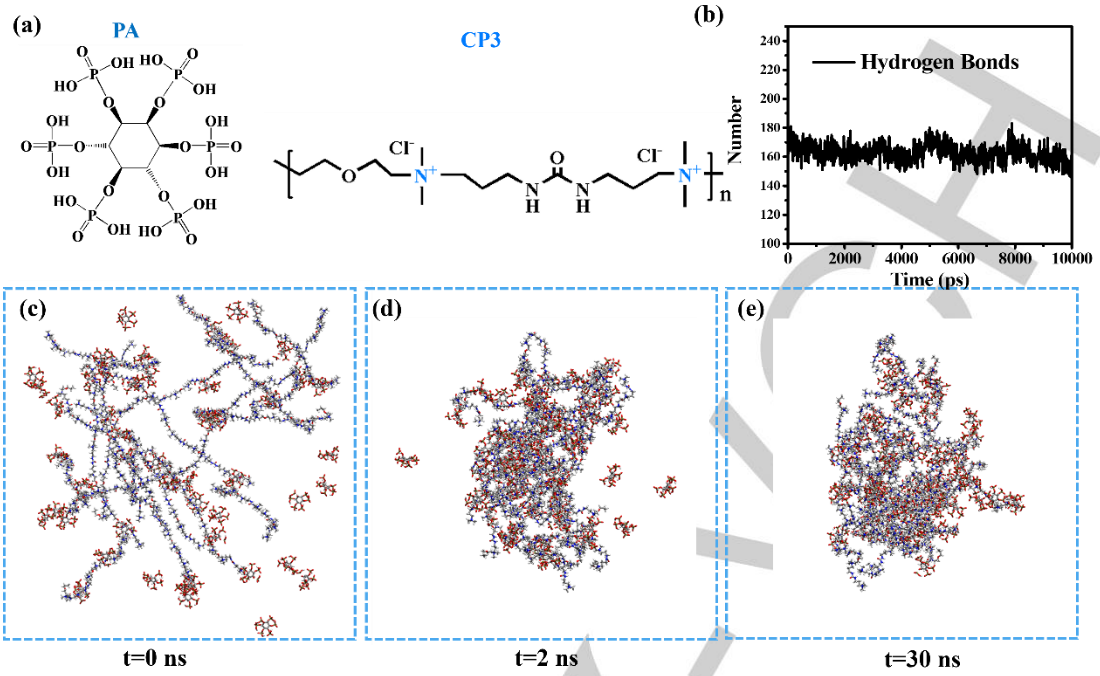

Figure S7. (a) Chemical structures of PA and CP3. (b) Number of hydrogen bonds of PA and CP3. Snapshots of the assembly of PA-CP3 at (c) 0, (d) 2 and (e) 30 ns in the MD simulation.

WILEY-VCH

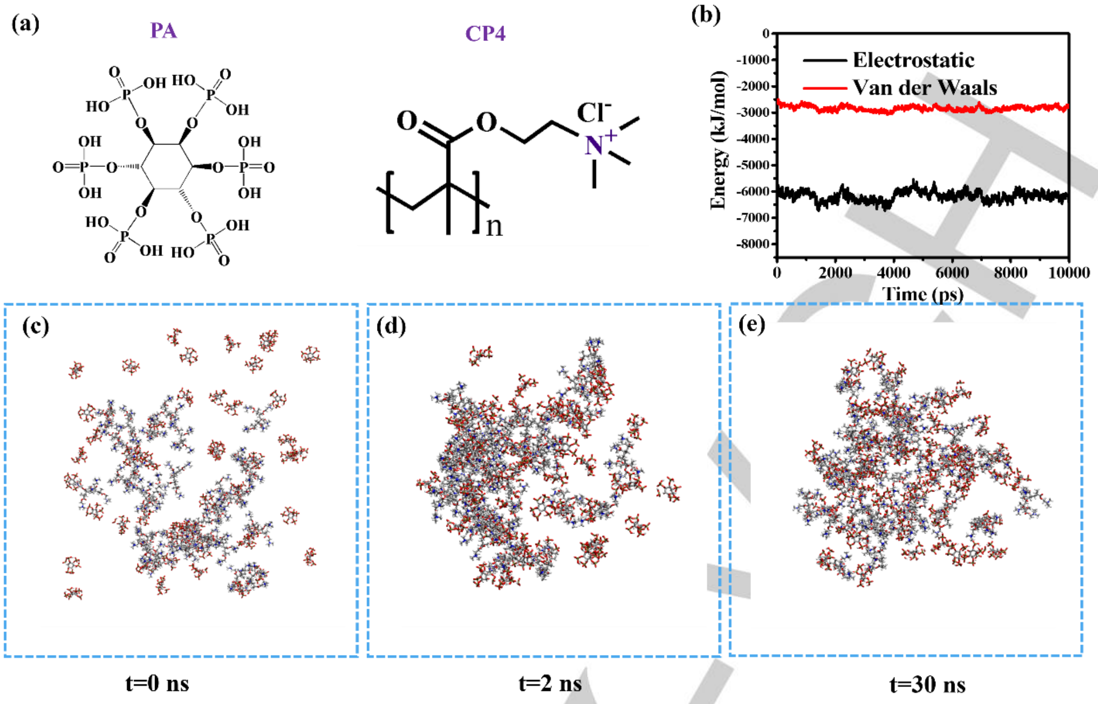

Figure S8. (a) Chemical structures of PA and CP4. (b) Electrostatic attraction and Van der Waals interaction energies of PA and CP4. Snapshots of the assembly of PA-CP4 at (c) 0, (d) 2 and (e) 30 ns in the MD simulation.

WILEY-VCH

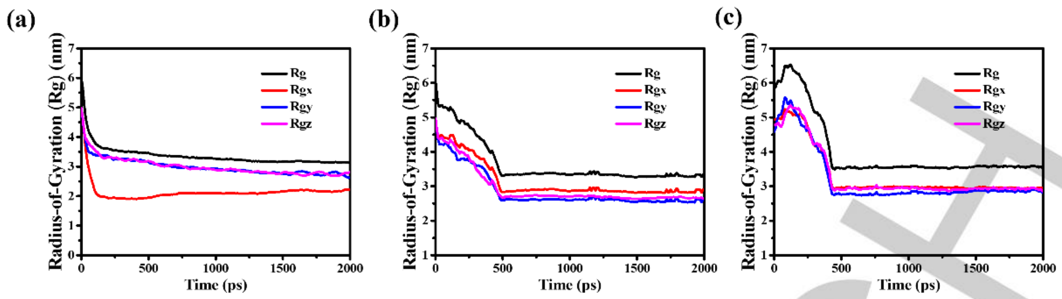

Figure S9. The radius of gyration (R_g) of (a) PA-CP2, (b) PA-CP3 and (c) PA-CP4 in the heating NPT conditions within 2 ns.

WILEY-VCH

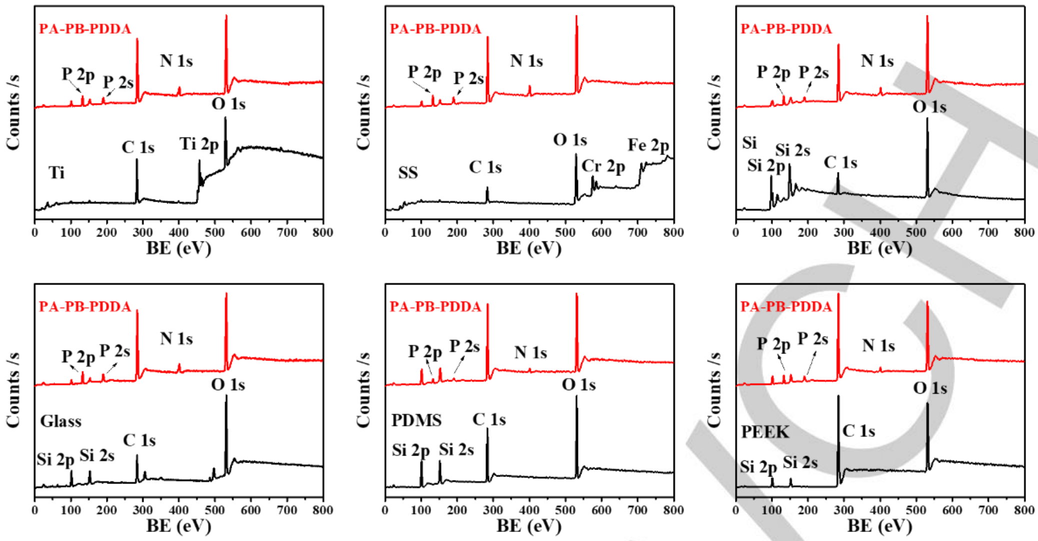

Figure S10. XPS wide-scan spectra of the Ti, SS, Si, glass, PDMS and PEEK surfaces before and after deposition of the PA-PB-PDDA coating.

WILEY-VCH

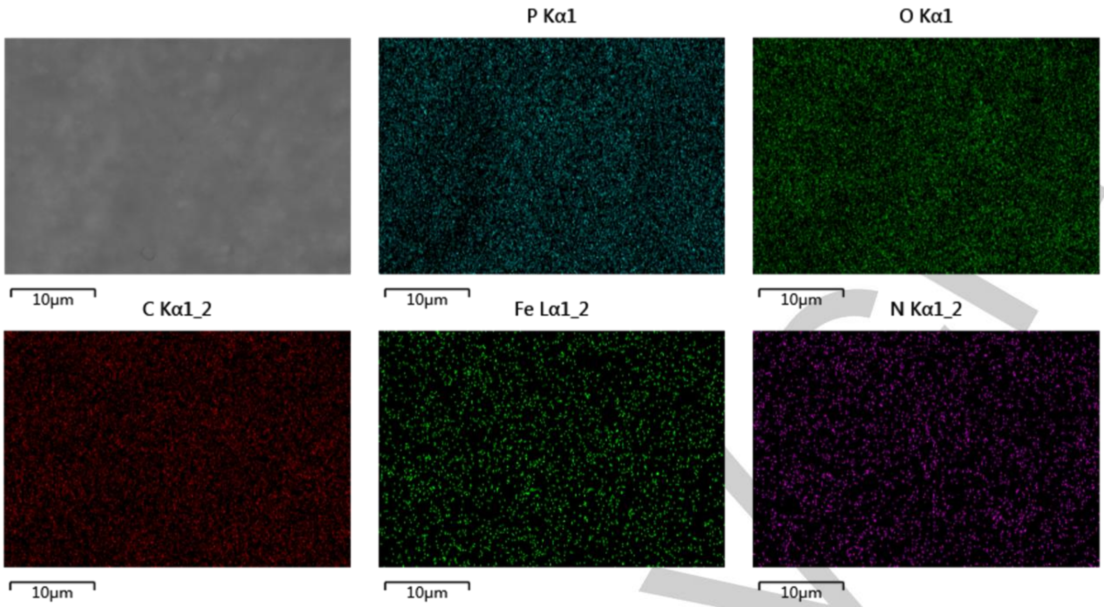

Figure S11. SEM and EDS mapping images of the PA-PB-PDDA coating on the Ti surfaces.

WILEY-VCH

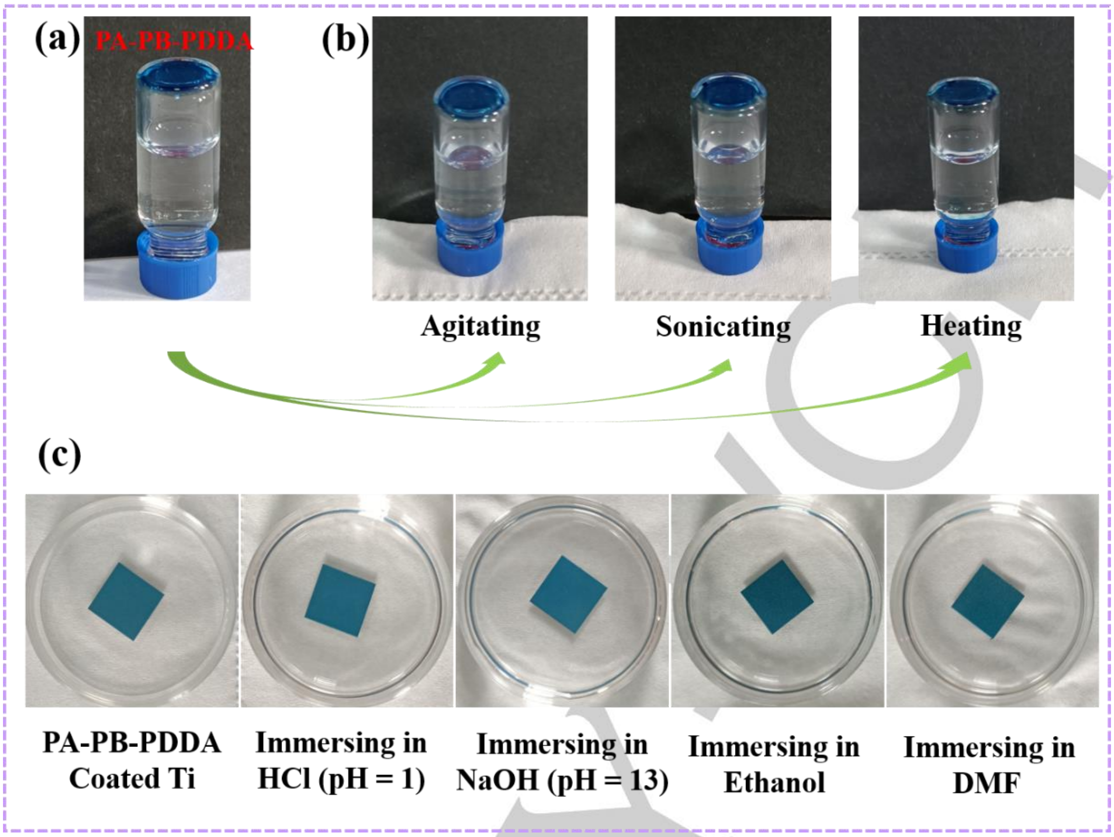

Figure S12. Stability of the PA-PB-PDDA coating. The PA-PB-PDDA layer adhered on the bottom of inverted glass vial (a) before and (b) after agitation by a vortex mixer for 5 min, sonicating for 30 min (40 Hz, 400 W), and heating at 80 ^{o}C for 30 min; (c) The photo images of the PA-PB-PDDA-coated Ti coupons before and after immersing in 5 1mL HCl (pH = 1), NaOH (pH = 13), ethanol and *N*,*N*-dimethylformamide (DMF) solutions (50% in water).

WILEY-VCH

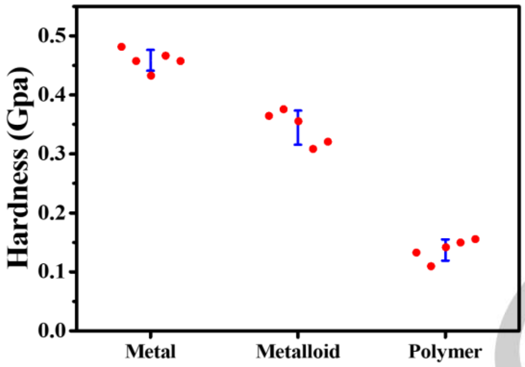

Figure S13. The hardness of PA-PB-PDDA coating on the Ti, glass and PDMS surfaces. The values were measured by Nanoindentation (G200, KLA Inc., USA), with surface approach velocity of 0.3 nm/s, and depth limit of 300 nm.

WILEY-VCH

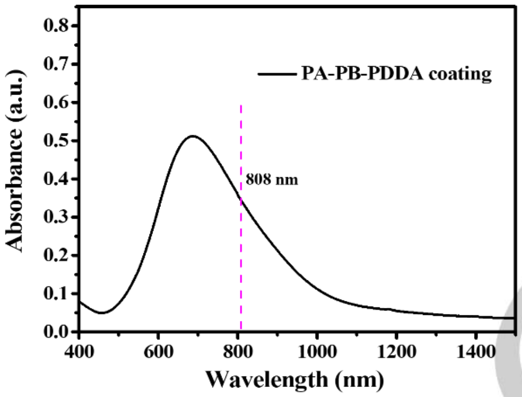

Figure S14: UV-visible absorption spectrum of PA-PB-PDDA coating on the PDMS substrate.

WILEY-VCH

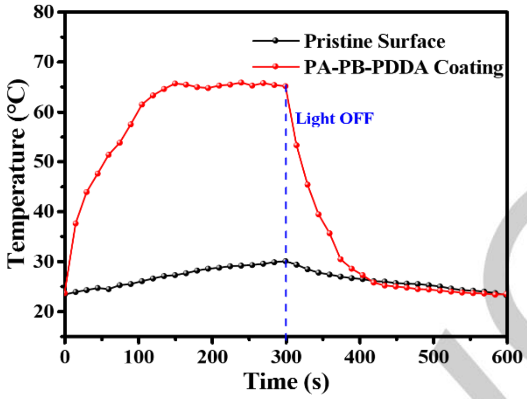

Figure S15. Time-dependent heating and cooling curves of the pristine PDMS and PDMS-PA-PB-PDDA substrates. The temperatures were recorded every 15 s.

WILEY-VCH

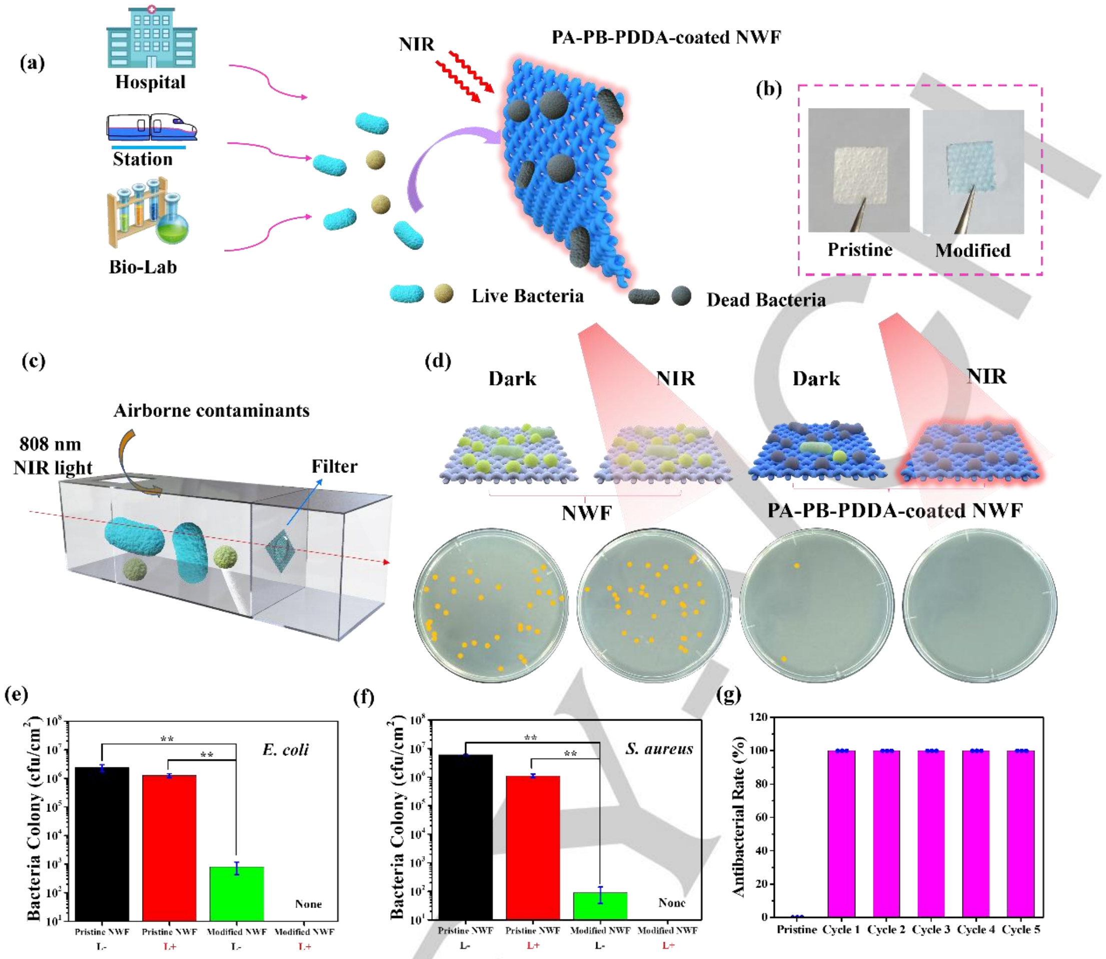

Figure S16. Antibacterial application of the PA-PB-PDDA-coated NWF filters. (a) Schematic representation of the PA-PB-PDDA-based NWF filter for air purification; (b) Photo images of the pristine and modified NWFs; (c) The ingenious air purification system; (d) Schematic illustration of the photothermal antibacterial ability of the modified NWF, and the tryptic soy broth (TSB)-agar plates inoculated with the detached bacteria from the contaminated NWF filters; Antibacterial performance of PA-PB-PDDA-modified NWF on (e) *E. coli* and (f) *S. aureus*; (g) Cyclic antibacterial test of PA-PB-PDDA-modified NWF against *S. aureus* under NIR irradiation. The error bars indicate the means ± SD ($n \geq 3$): $^{**}p < 0.01$.

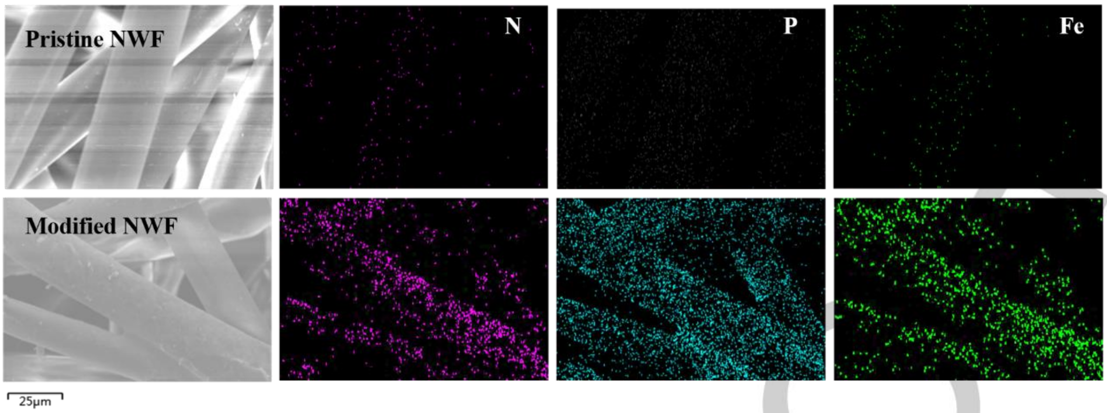

Figure S17. SEM and EDS mapping (N, P and Fe elements) of the NWF filter before and after the deposition of PA-PB-PDDA coating. Scale bar = 25 μm.

WILEY-VCH

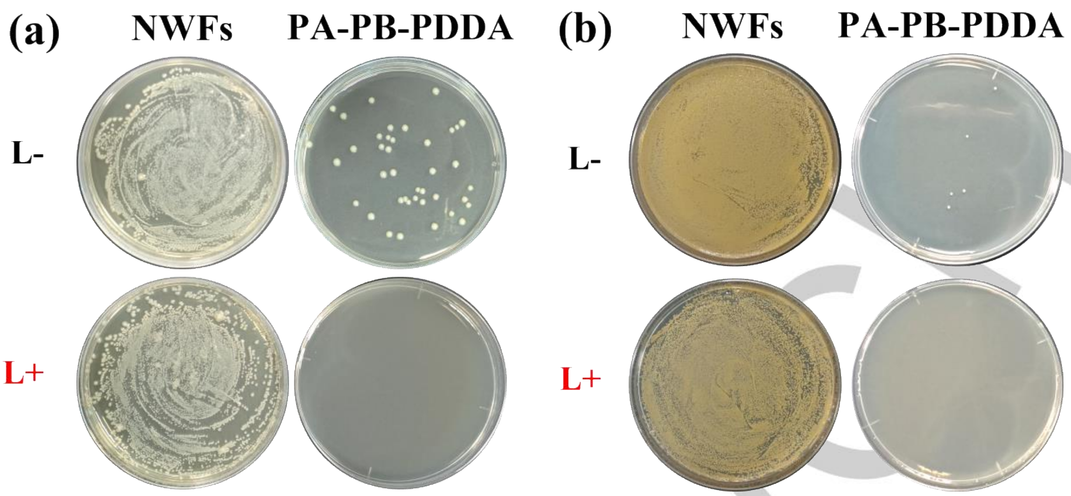

Figure S18. The antibacterial performance of the pristine and PA-PB-PDDA-coated NWFs upon immersion in the bacterial suspension (10^7 cfu/mL) in the presence (L+)/absence (L-) of NIR irradiation. The TSB-agar plates inoculated with the detached (a) *E. coli* and (b) *S. aureus* from the pristine and modified NWFs.

WILEY-VCH

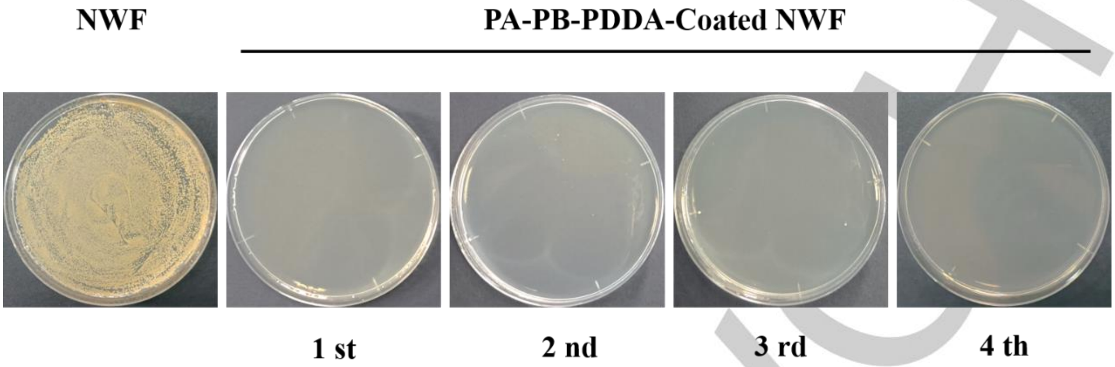

Figure S19. The antibacterial performance of the PA-PB-PDDA-coated NWF under NIR irradiation after repeated usage.

WILEY-VCH

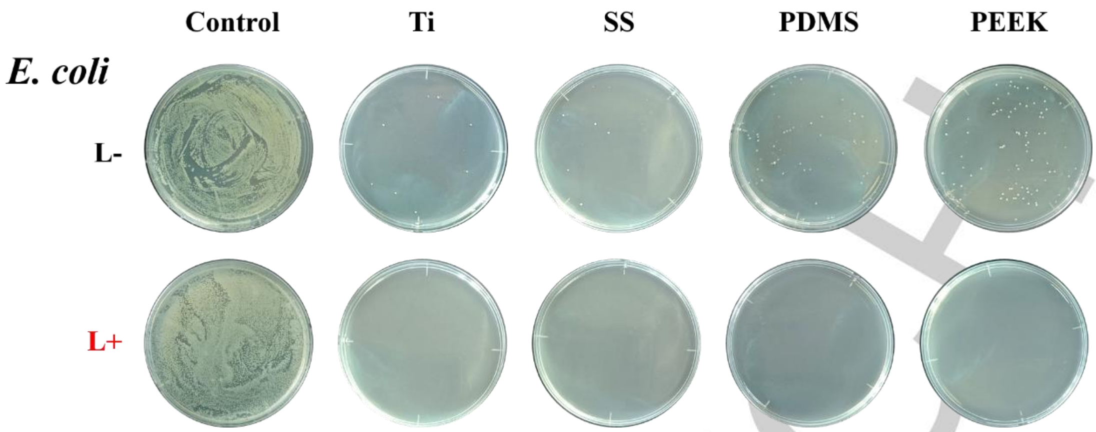

Figure S20. The TSB-agar plates inoculated with the detached *E. coli* from the pristine and modified biomaterial surfaces. Similar bactericidal properties were observed on various substrates under NIR irradiation.

WILEY-VCH

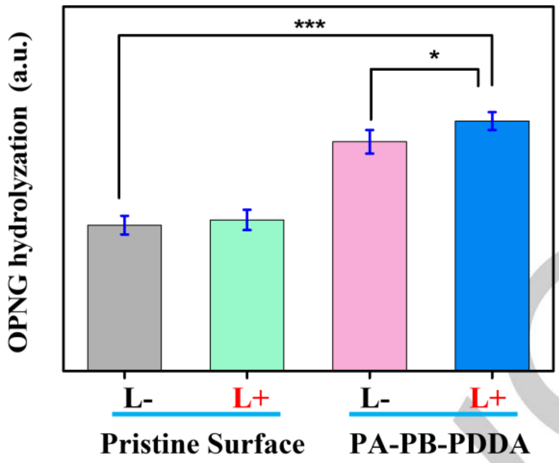

Figure S21. ONPG hydrolysis of *E. coli* after incubation with the pristine and modified substrate surfaces in the presence/absence of NIR irradiation.

WILEY-VCH

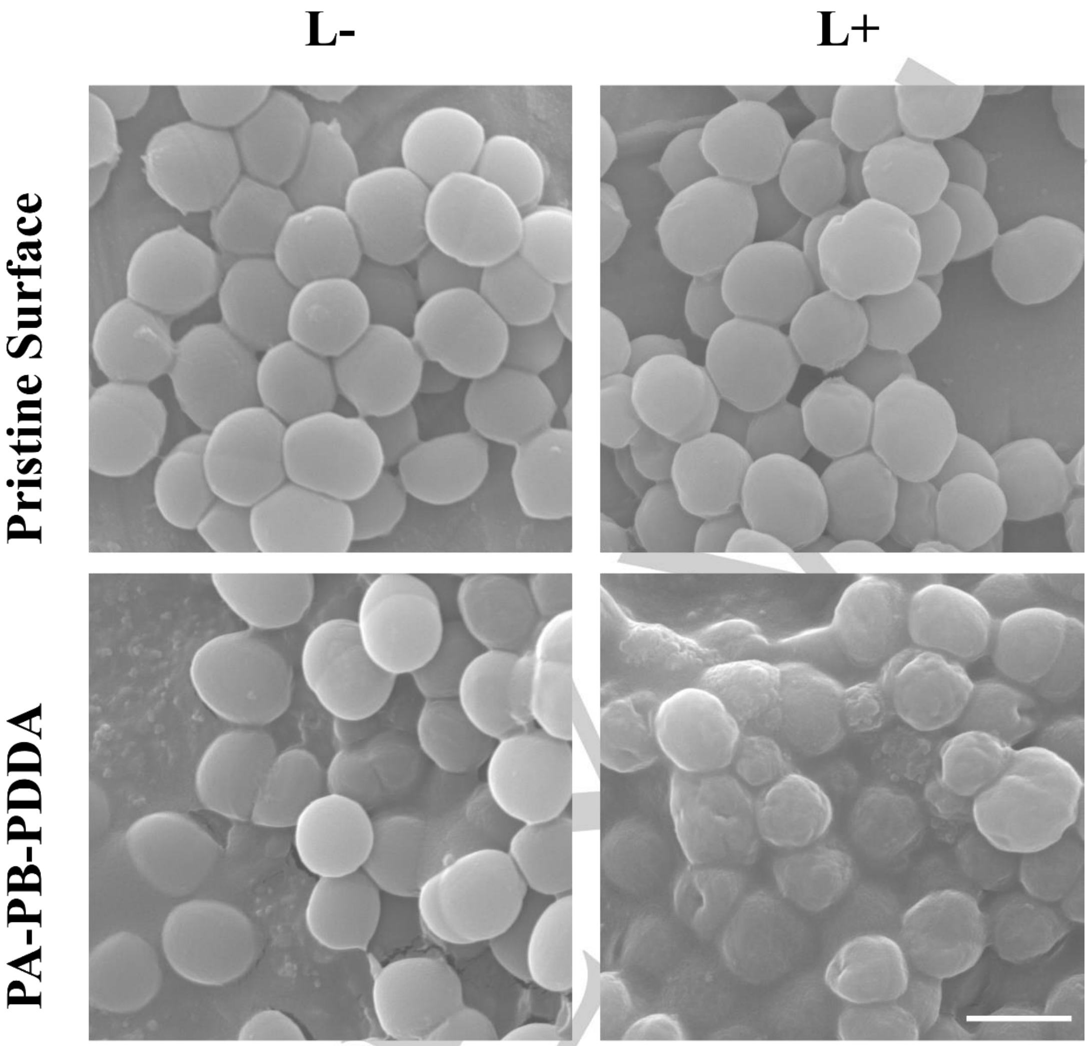

Figure S22. FESEM images of *S. aureus* adhered on the pristine and PA-PB-PDDA-modified SS surfaces with (L+) or without (L-) NIR treatment (scale bar = 1 μm).

WILEY

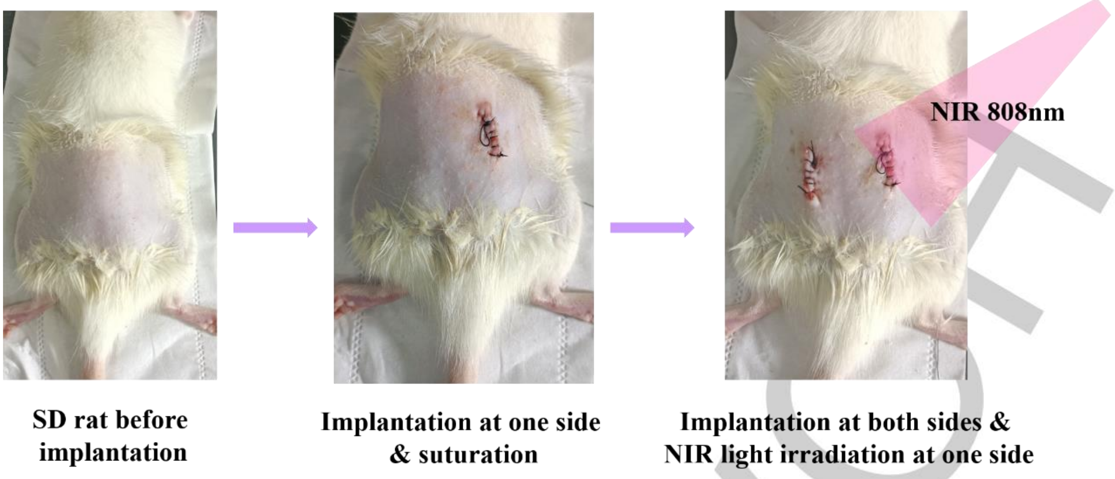

Figure S23. The subcutaneous implantation model. The SD rat was anesthetized, and the dorsal section of the rat was shaved and sterilized. The skin was then incised (about 1.5 cm in length), followed by the implantation of the pristine SS and SS-PA-PB-PDDA substrates (10 mm × 10 mm) beneath the skin. The implants were inoculated with 15 µL *S. aureus* suspension at a concentration of 1 × 10^7 CFU/mL, and the skins were stitched.

WILEY-VCH

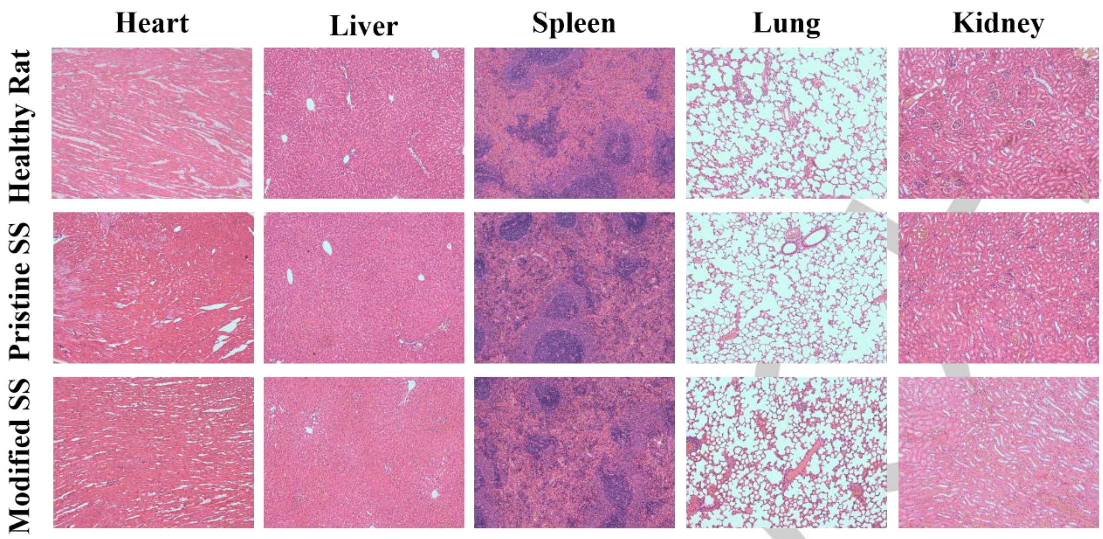

Figure S24. The H&E staining images of heart, liver, spleen, lung and kidney of the healthy, pristine SS-implanted, and modified SS-implanted SD rats.

WILEY-VCH

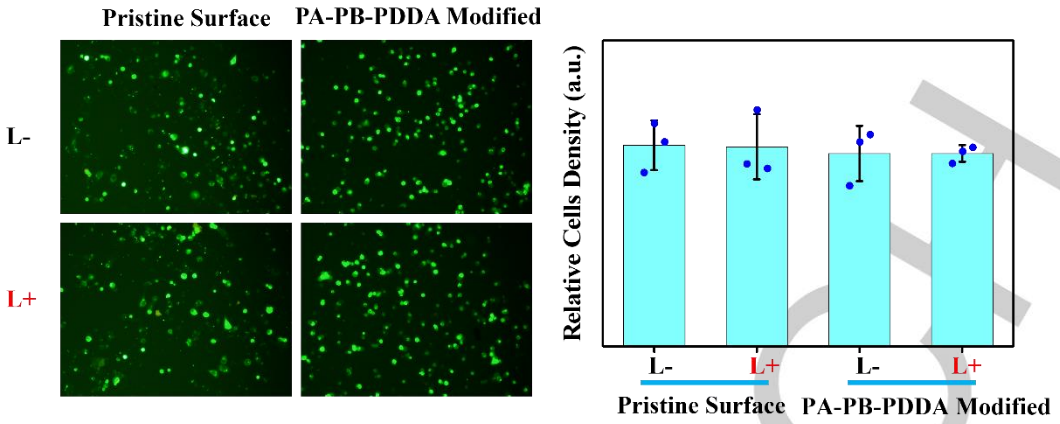

Figure S25. MC3T3-E1 cells cultured on the pristine Ti and Ti-PA-PB-PDDA surfaces in a 24-well plate, and stained by Cell Plasma Membrane Staining Kit with DiO (Green Fluorescence).

WILEY-VCH

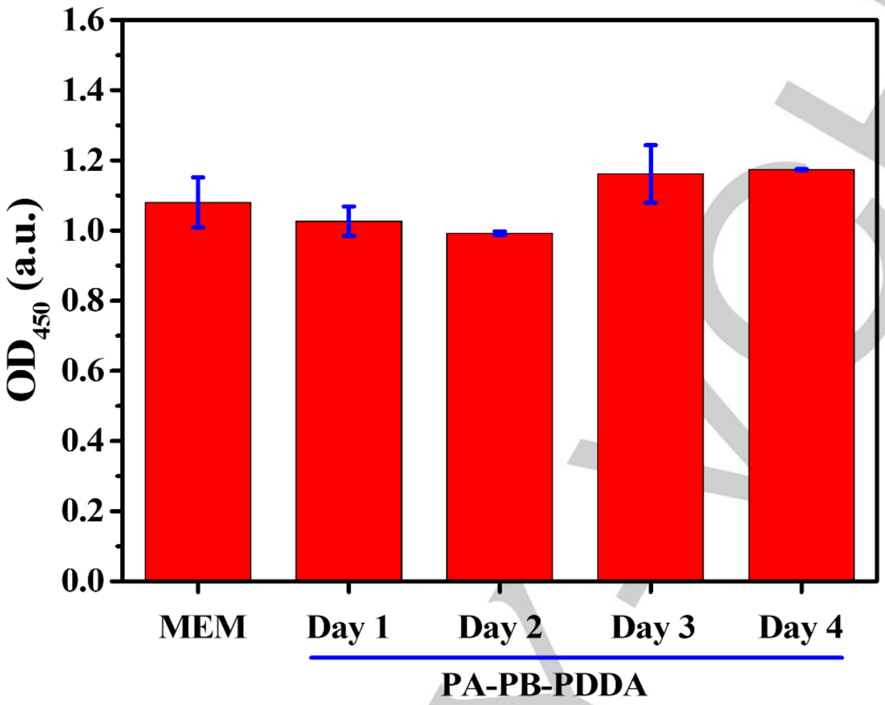

Figure S26. Cytotoxicity of the Ti-PA-PB-PDDA substrates toward MC3T3-E1 cells, evaluated by the CCK-8 assay.

WILEY-VCH

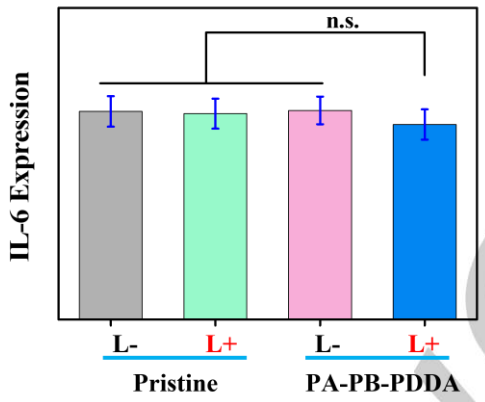

Figure S27. Production of inflammatory cytokines (IL-6) of RAW264.7 cells after incubation with PBS (pristine) and the PA-PB-PDDA composite, as measured by the IL-6 ELISA Kit.

WILEY-VCH

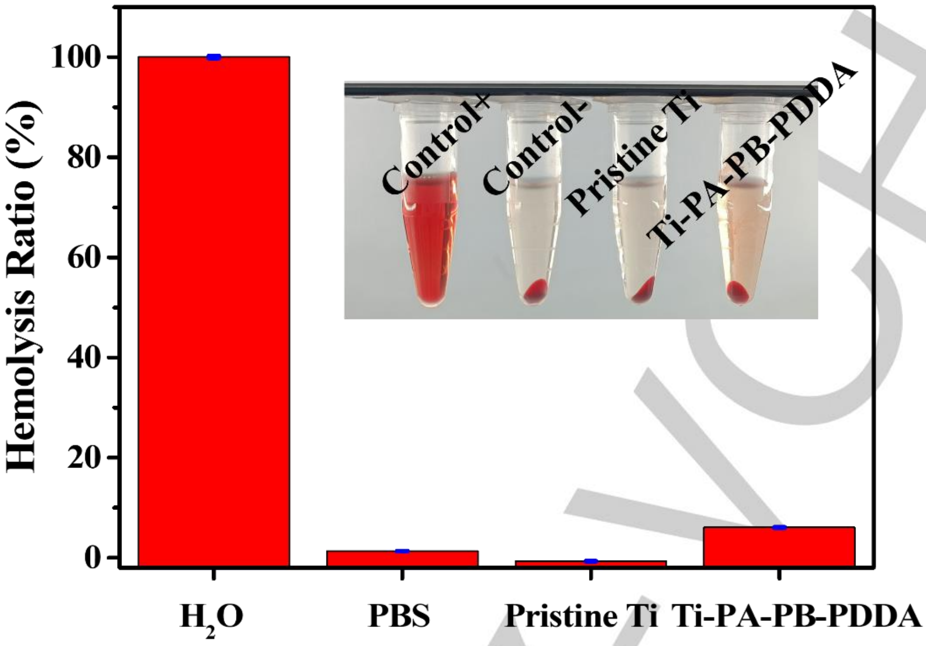

Figure S28. Hemolysis assay of the pristine Ti and Ti-PA-PB-PDDA surfaces. The pristine and modified Ti substrates were immersed into the suspension of red blood cells (RBCs) at 37 °C for 2 h. PBS and deionized water were used as the negative and positive controls, respectively.

Reference

[1] G. Bussi, D. Donadio, M. Parrinello, *J Chem Phys* **2007**, 126.

[2] H. J. C. Berendsen, J. P. M. Postma, W. F. Vangunsteren, A. Dinola, J. R. Haak, *J Chem Phys* **1984**, 81, 3684.

[3] T. Darden, D. York, L. Pedersen, *J Chem Phys* **1993**, 98, 10089.

[4] D. Van der Spoel, E. Lindahl, B. Hess, G. Groenhof, A. E. Mark, H. J. C. Berendsen, *J Comput Chem* **2005**, 26, 1701.

[5] W. Humphrey, A. Dalke, K. Schulten, *J Mol Graph Model* **1996**, 14, 33.
